# Microdroplet Stabilization Enabled Direct Mass Spectrometric Identification of Electrogenerated Transient Carbocation Intermediates

**DOI:** 10.1002/advs.202518157

**Published:** 2025-12-08

**Authors:** Guo‐Shan Zhu, Ren‐Jie Hui, Jun Hu

**Affiliations:** ^1^ School of Life Sciences and Health Engineering Jiangnan University Wuxi 214122 China

**Keywords:** carbocation intermediate, C─H amination, electrosynthesis, mass spectrometry, microdroplet

## Abstract

The electrochemically generated carbocations are pivotal yet elusive intermediates in electrosynthesis. By coupling nanoelectrospray ionization with a tip‐confined electrochemical microreactor, the ultrafast transfer of the electrogenerated species into the charged microdroplets is achieved. Interestingly, the highly reactive carbocations become abnormally inert in the electrosprayed microdroplets, and made a survival throughout the entire lifespans of the microdroplets. By taking full advantage of the newfangled stabilizing effect of the microdroplets, the direct mass spectrometric identification of the electrogenerated transient benzylic and phenylic carbocations, whose half‐lives in bulk solutions are on the nanosecond level is achieved. The detailed paths of the carbocation‐driven C(sp^3^)–H and C(sp^2^)–H aminations are also revealed by the stepwise evaluation of their initial electrochemical generation and their ultrafast succedent chemical transformations. With its high sensitivity, excellent specificity, and minimal time delay, the wide applications of the in situ electrochemical mass spectrometry is anticipated as a powerful tool for not only the identification of previously unseen intermediates but also the in‐depth exploration of the intricate electrochemical transformations.

## Graphical abstract

The in situ mass spectrometric identification of the electrogenerated benzylic and phenylic carbocations, whose half‐lives in bulk solutions are on the nanosecond scale, is achieved by taking advantage of the unique stabilization effect of the electrosprayed microdroplets.

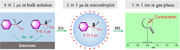

## 1 Introduction

In organic electrosynthesis, highly reactive intermediates can be conveniently generated under very mild conditions through single‐electron transfer (SET) within the electrode‐solution interface, enabling diverse transformations that were previously considered challenging or even impossible.^[^ ^1^, ^2^ ^]^ Compared with conventional approaches, electrochemical synthesis is featured by high atom economy and environmental friendliness, as it employs the clean electrons to drive the desired redox transformations and thus avoids the use of hazardous oxidants/reductants.^[^ ^3^, ^4^ ^]^ Moreover, by virtue of the fine‐tuning of applied potential, it also allows the precise control over the generation of certain intermediates to maximize the selectivity and yield.^[^ ^5^, ^6^, ^7^ ^]^ Among these intermediates, carbocations play pivotal roles in many electrochemical transformations, especially the C─C, C─N, C─O, C─S bond formations.^[^ ^8^, ^9^, ^10^, ^11^, ^12^, ^13^, ^14^, ^15^ ^]^ Notably, the intrinsic electron‐deficient nature of carbocations makes them extremely reactive toward nucleophiles (including the common solvents), resulting in their transient in‐solution half‐lives as well as challenges in validations of their actual existences and exact roles. For instance, the half‐lives of the benzylic and phenylic carbocations in acetonitrile water are estimated to be ≈0.7 ns and 3–20 ns, respectively.^[^ ^16^, ^17^ ^]^ Although the recognition of carbocations as reactive intermediates can be dated back to 1920s,^[^ ^18^, ^19^, ^20^ ^]^ the direct capture and identification of the electrogenerated transient carbocation intermediates still remains elusive.

In the past decades, various advanced analytical techniques, such as low‐temperature nuclear magnetic resonance (NMR) spectroscopy,^[^ ^21^ ^]^ gas‐phase ion spectroscopy,^[^ ^22^, ^23^ ^]^ and transient absorption spectroscopy (TAS),^[^ ^24^ ^]^ have been devoted to the mechanismic understanding of carbocation‐mediated reactions. Unfortunately, the restrictions of these methods in neither the response time nor the chemical specificity, combined with their complicated instrumental implementation with electrochemistry, have frequently led to failures in simultaneously characterizing the various chemical species involved in an electrochemical transformation, especially the short‐lived, low‐concentration, and often fragile carbocation intermediates. Compared with the above techniques, mass spectrometry (MS) is a powerful tool for identifying the diverse species involved in an electrochemical reaction. The in situ combination of an electrochemical cell with mass spectrometry permits the low‐delay detection of the short‐lived intermediates.^[^ ^25^, ^26^, ^27^, ^28^, ^29^, ^30^, ^31^ ^]^ For example, Zare et al recently built a waterwheel‐shaped working electrode to allow the coupling of electrochemistry with desorption electrospray ionization mass spectrometry (DESI‐MS). This novel method achieved the direct identification of many electrogenerated reactive intermediates, with half‐lives ranging from milli‐ to even micro‐seconds.^[^ ^25^, ^26^, ^27^ ^]^ More recently, the elegant work by Banerjee et al reported the unique stabilizing effect of water microdroplets, in which the carbocations become abnormally inert and could make a survival throughout the whole lifespan of the diminishing microdroplets.^[^ ^32^, ^33^, ^34^ ^]^ Inspired by this, we thus propose the feasibility of directly detecting the previously challenging electrogenerated carbocations (whose half‐lives in bulk solutions are typically in nano‐ to pico‐second range) using electrospray ionization mass spectrometry (ESI‐MS). However, the rapid transfer of the newly electrogenerated carbocations into the stabilizing microdroplets is still a prerequisite for the successful detection of these transient intermediates.^[^ ^35^ ^]^

**Figure 1:**
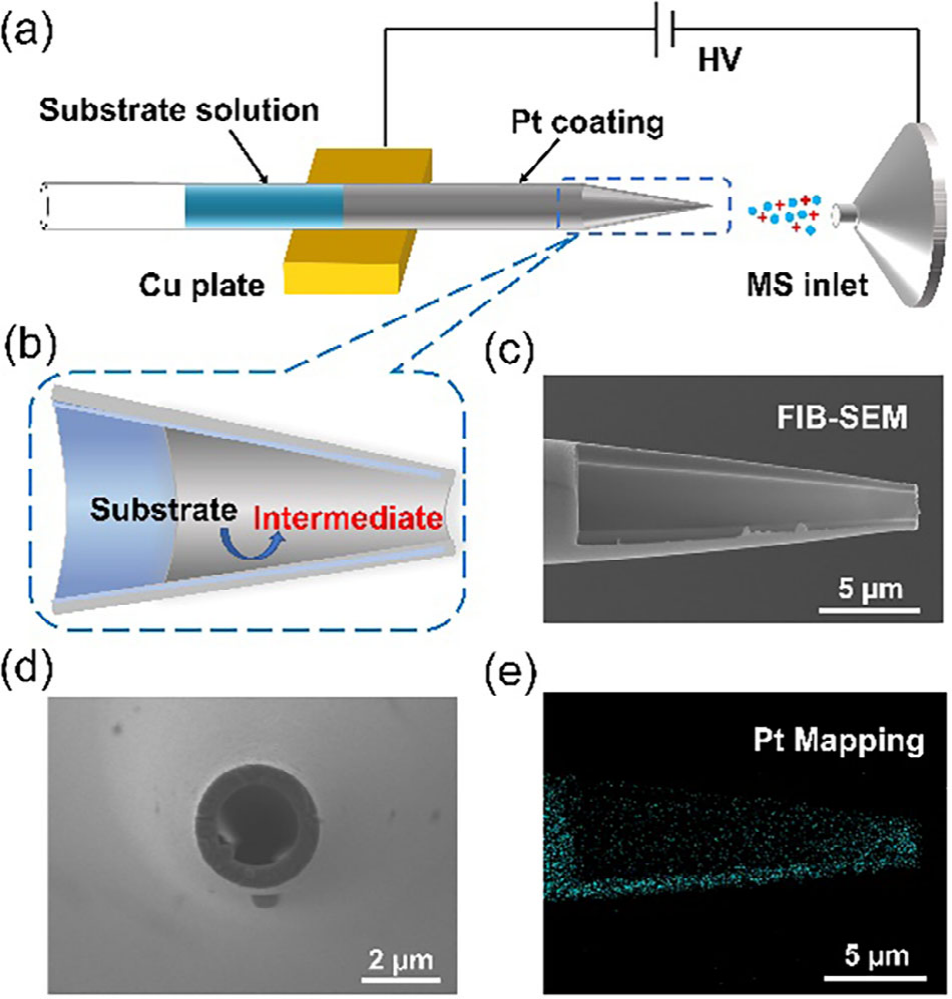
a) Schematic diagram of in situ electrochemical mass spectrometry, b) Schematic diagram of hybrid ultramicroelectrode/ion emitter, c) FIB‐SEM image of a Pt‐decorated micropipette, d) SEM image of a Pt‐decorated micropipette (top view), and e) element mapping image of a Pt‐decorated micropipette.

In this work, we presented the rapid mass spectrometric identification of the electrogenerated transient carbocation intermediates, achieved by the in situ combination of a tip‐confined electrochemical microreactor with nanoelectrospray ionization (nESI). As illustrated in **Figure** **1**a and S1 (Supporting Information), an ultramicroelectrode was precisely constructed into a tapered glass capillary by metal deposition (Pt, ≈100 nm in thickness). Crucially, the metal layer is also continuously deposited onto the outer surface of the capillary, enabling direct electrical contact for high‐voltage implementation. The micro‐sized glass capillary kept hollow after the Pt decoration, thus maintaining the function of an ion emitter (see Figure 1c,e; Figure S2, Supporting Information for the characterizations). The simultaneous activation of the electrooxidation of a substrate and the positive‐ion electrospray ionization can thus be achieved by applying a positive high voltage, and vice versa.^[^ ^36^, ^37^, ^38^, ^39^, ^40^, ^41^ ^]^ Importantly, the Pt thin layer inside the tapered tip was only a few microns in length (≈5 µm, see the element mapping in Figure 1e). The electrogenerated species can thus traverse an extremely short distance to the Taylor cone, at which the charged microdroplets are sprayed out. Key to the successful mass spectrometric detection of the electrogenerated transient carbocations is the ultrafast transfer (<1 µs) of these intermediates into the microdroplets, in which they are stabilized until the final release of corresponding gaseous ions for the mass spectrometric detection.

## 2 Results and Discussion

### 2.1 Identification of the Benzylic Carbocation Intermediates Produced in Electrochemical C(sp^3^)–H Aminations

**Figure 2:**
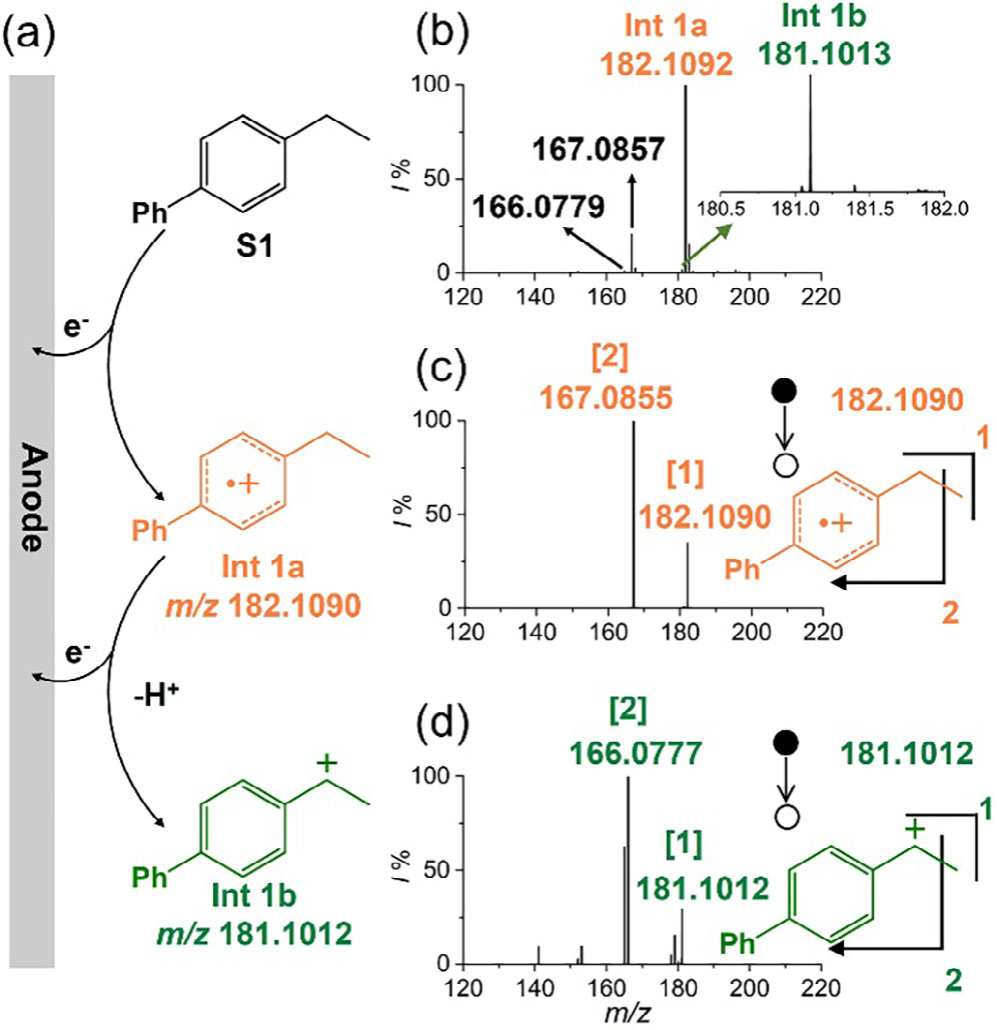
a) Proposed mechanism for the successive two‐electron electrooxidation of 4‐ethylbiphenyl, b) Mass spectra of 4‐ethylbiphenyl obtained using a hybrid ultramicroelectrode/ion emitter, c) MS/MS of **Int 1a**, and d) MS/MS of **Int 1b**.

To validate the capacity of this method in the detection of the electrogenerated carbocations, we first selected the classic benzylic carbocation as a proof‐of‐concept test. The electrochemical generation of benzylic carbocations from easily available starting materials has been recently reported by Xu et al.^[^ ^9^ ^]^ Further nucleophilic trapping of the benzylic carbocations by amine leads to the site‐selective C(sp^3^)–H amination under mild conditions, without the need for transition‐metal catalysts and external chemical oxidants.^[^ ^9^ ^]^ As illustrated in **Figure** **2**a and 4‐ethylbiphenyl (**S1**) was proposed that first proceeds an initial SET oxidation on the anode, generating a short‐lived cationic radical (**Int 1a**) that undergoes a further one‐electron, one‐proton transfer oxidation to form the transient benzylic carbocation (**Int 1b**). Figure 2b showed the mass spectrum obtained from DCE/HFIP (1:1) containing 1 mM 4‐ethylbiphenyl using a hybrid ultramicroelectrode/ion emitter. According to the accurate mass measured by a high‐resolution Orbitrap mass spectrometer, the electrochemical formation of **Int 1a** was confirmed by the appearance of a mass peak at *m/z* 182.1092 ([C_14_H_14_]^●+^, theoretical *m/z* 182.1090, *∆m* = 0.2 mDa). To our delight, the expected benzyl carbocation was also confirmed by a signal at *m/z* 181.1013 ([C_14_H_13_]^+^, theoretical *m/z* 181.1012, *∆m* = 0.1 mDa).

It is also noteworthy that an apparent signal located at *m/z* 167.0857, as well as a minor signal at *m/z* 166.0779, were observed. This might be rationally explained by the structural frangibility of the two intermediates, which might have undergone in‐source fragmentation in the ionization region. In fact, upon the standard collision‐induced dissociation (CID), a major fragment located at *m/z* 167.0855 was produced from **Int 1a**, assigned to the neutral loss of a ^●^CH_3_ (*Δm* = 15.0235 Da, matches exactly with the theoretical mass of ^●^CH_3_, see Figure 2c). This fragmentation pattern is in line with the structure of **Int 1a**, which losses a ^●^CH_3_ and experiences a ring expansion rearrangement to the more stable tropylium cation.^[^ ^42^ ^]^ Similarly, the minor signal at *m/z* 166.0779 in Figure 2b was believed to originate from **Int 1b**, as evidenced by tandem mass spectrometry (MS/MS) shown in Figure 2d. The parent ion, referring to **Int 1b,** mainly fragmented into a daughter ion that was located at *m/z* 166.0777 by losing a ^●^CH_3_, accompanied by the less abundant fragments at *m/z* 165.0700, *m/z* 152.0622, and *m/z* 141.0699 through the loss of CH_4_, ^●^C_2_H_5_, and C_3_H_4_, respectively. The above results proved that, although both **Int 1a** and **Int 1b** are short‐lived and unstable, their successful mass spectrometric identification could be achieved by taking advantage of the dual‐function ultramicroelectrode/ion emitter. Moreover, the unambiguous detection of nanosecond‐lived **Int 1b** in DCE/HFIP also demonstrated that the stabilizing effect of microdroplets toward carbocations is not limited to the aqueous system.^[^ ^32^, ^33^, ^34^ ^]^ We also note that the relative ion intensity of the carbocation intermediate could be significantly increased by adding acid (e.g., AcOH) into the solvent system (Figure S3, Supporting Information). This confirmed the acid‐induced stabilization of the transient carbocations within the sprayed microdroplets.

To further evaluate the usability of our electrochemical mass spectrometry (EC‐MS), the reaction substrates were extended to 14 benzyl derivatives. On the basis of the high mass accuracies (Table S1 and Figure S4, Supporting Information) as well as the characteristic patterns of MS/MS (Figure S5, Supporting Information), the corresponding carbocations electrogenerated from benzyl substrates with diverse substituent groups were all successfully detected, exhibiting the great applicability of this in situ EC‐MS method.

**Figure 3:**
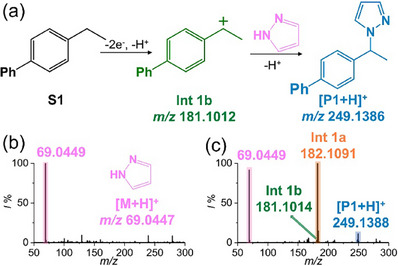
a) Proposed reaction mechanism for the nucleophilic trapping of the benzylic carbocation by pyrazole, b) Mass spectra obtained using a bare ion emitter, and c) Mass spectra obtained using a hybrid ultramicroelectrode/ion emitter.

The electrochemical C─H/N─H cross‐couplings represent an ideal approach for constructing the C─N bonds, because of the high atom economy and superior environmental friendliness.^[^ ^1^, ^2^, ^3^, ^4^ ^]^ The carbocation‐mediated C─N bond reaction was thus explored by adding an effective nucleophile, i.e., pyrazole, into the solution (see **Figure** **3**a for the reaction mechanism proposed by Xu et al).^[^ ^9^ ^]^ A control experiment was first conducted using a bare ion emitter loaded with a solution containing 4‐ethylbiphenyl (1 mm) and pyrazole (4 mm), see Figure S6a (Supporting Information) for the experimental setup. By placing the Pt electrode at > 4 cm away from the tip front, only protonated ion of pyrazole was observed at *m/z* 69.0445 ([C_3_H_4_N_2_+H]^+^, theoretical *m/z* 69.0447, *∆m* = −0.2 mDa, see Figure 3b; Figure S6b, Supporting Information). The absence of signals referring to the targeted product (**P1**) confirmed the essential role of the anodic oxidation. This also excluded the spontaneous oxidation of the substrate (e.g., **S1**) in the sprayed microdroplets by the inherent ultrahigh electric field at the microdroplet's interface (up to 10^9^ V m^−1^).^[^ ^43^, ^44^ ^]^ This might be explained by the limited microdroplet reaction time (typically a few to tens of microseconds), due to the small dimensions of the microdroplets in nESI.^[^ ^45^, ^46^ ^]^ The same solution was then analyzed using an ultramicroelectrode/ion emitter. Fortunately, apart from the mass peaks of benzyl intermediates (i.e., **Int 1a** and **Int 1b**), the formation of the expected product was also evidenced by a signal at *m/z* 249.1388 ([**P1**+H] ^+^, theoretical *m/z* 249.1386, *∆m* = 0.2 mDa).

It is worth noting that the tip‐based microreactor in this work is superior in achieving the mass spectrometric detection of the electrogenerated transient intermediates. Although the signal referring to the final product (**P1**) was observed by placing the Pt electrode at the very front, both **Int 1a** and **Int 1b** escaped the mass spectrometric detection when using the conventional wire‐in‐a‐capillary method (Figure S6c,d, Supporting Information), due to the relatively long transfer distance (≫100 µm). Our previously developed bipolar electrode (BPE)‐based method is featured by its sensitive and low‐delay detection of the electrogenerated species.^[^ ^28^, ^29^ ^]^ But it also failed in the detection of the expected intermediates as well as the final products (see Figures S6e,f, Supporting Information), suggesting no substrate electrooxidation has occurred. This might be attributed to the insufficient induced potential on the wireless BPE (typically <1 V vs Ag/AgCl,^[^ ^28^, ^29^ ^]^ while the required oxidation potential for producing the carbocation intermediate from **S1** is ≈2.0 V vs Ag/AgCl).^[^ ^9^ ^]^

Notably, the expected products of other benzyl derivatives with pyrazole were all identified on the basis of the accurate mass measurements (Table S2 and Figure s7, Supporting Information) as well as the tandem mass spectrometry (Figure S8, Supporting Information). In addition, the reactivities of other nucleophiles, including pyrazole derivatives (Table S3 and Figures S9 and S10, Supporting Information), triazole derivatives (Table S4 and Figures S11 and S12, Supporting Information), pyridine derivatives (Table S5 and Figures S13 and S14, Supporting Information) and miscellaneous nucleophiles (Table S6 and Figures S15 and S16, Supporting Information), were also tested, with the successful identifications of all involved intermediates and final products.

### 2.2 Identification of the Phenylic Carbocation Intermediates Produced in Electrochemical C(sp^2^)–H Aminations

**Figure 4:**
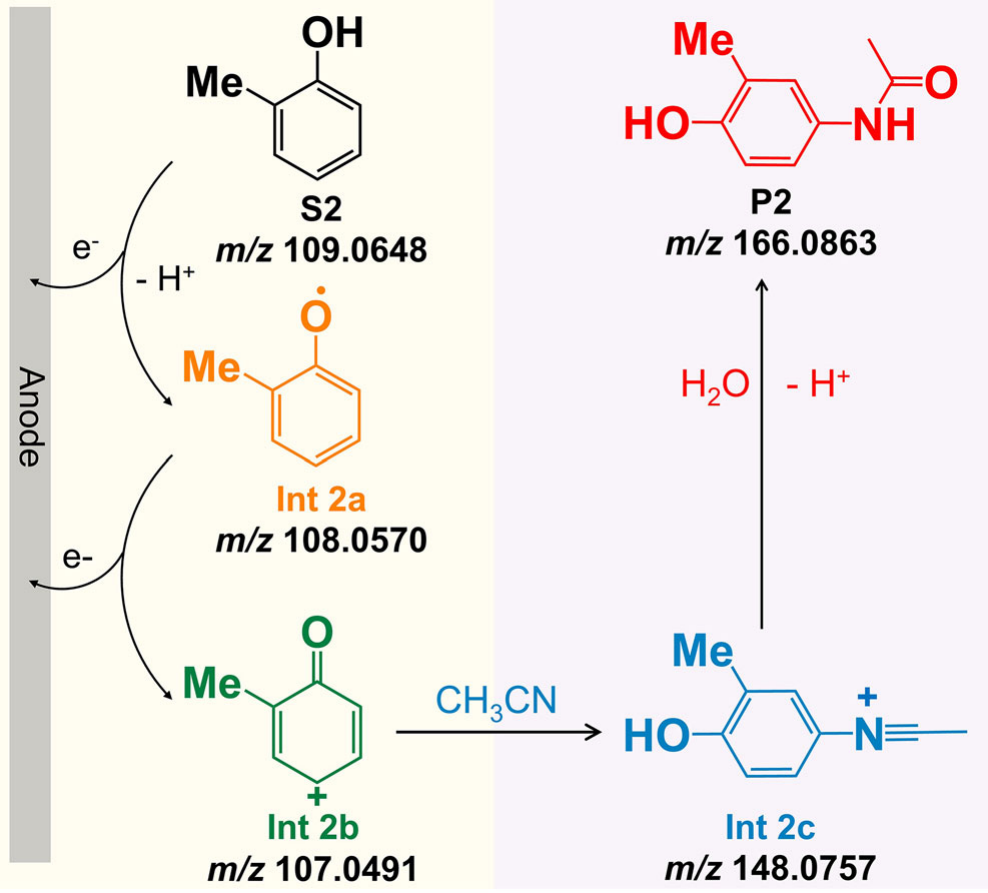
Proposed reaction pathway for the electrochemical Ritter‐type C(sp_2_)‐H amination of an unprotected phenol derivative.

With the success in benzylic carbocations, we are encouraged to the identification of the challenging electrogenerated phenylic carbocations. The regioselective Ritter reaction for the one‐step one‐pot synthesis of paracetamol from unprotected phenol via the electrochemical C(sp^2^)‐H activation was recently reported by Taily and co‐workers^[^ ^47^ ^]^ proposing a phenylic carbocation‐mediated reaction pathway as shown in **Figure** **4**a. In brief, the successive two‐electron oxidation of a phenol derivative produces the corresponding transient carbocation intermediate (**Int 2b**), passing through the formation of a short‐lived radical intermediate (**Int 2a**). Once produced, the highly reactive **Int 2b** undergoes the electrophilic addition with acetonitrile to form a nitrilium ion intermediate (**Int 2c**, i.e., the Ritter intermediate), followed by the hydrolysis of **Int 2c** to afford the final amide product. Unfortunately, no direct experimental evidence is currently available to support the actual existence as well as the proposed transformations of these intermediates.

**Figure 5:**
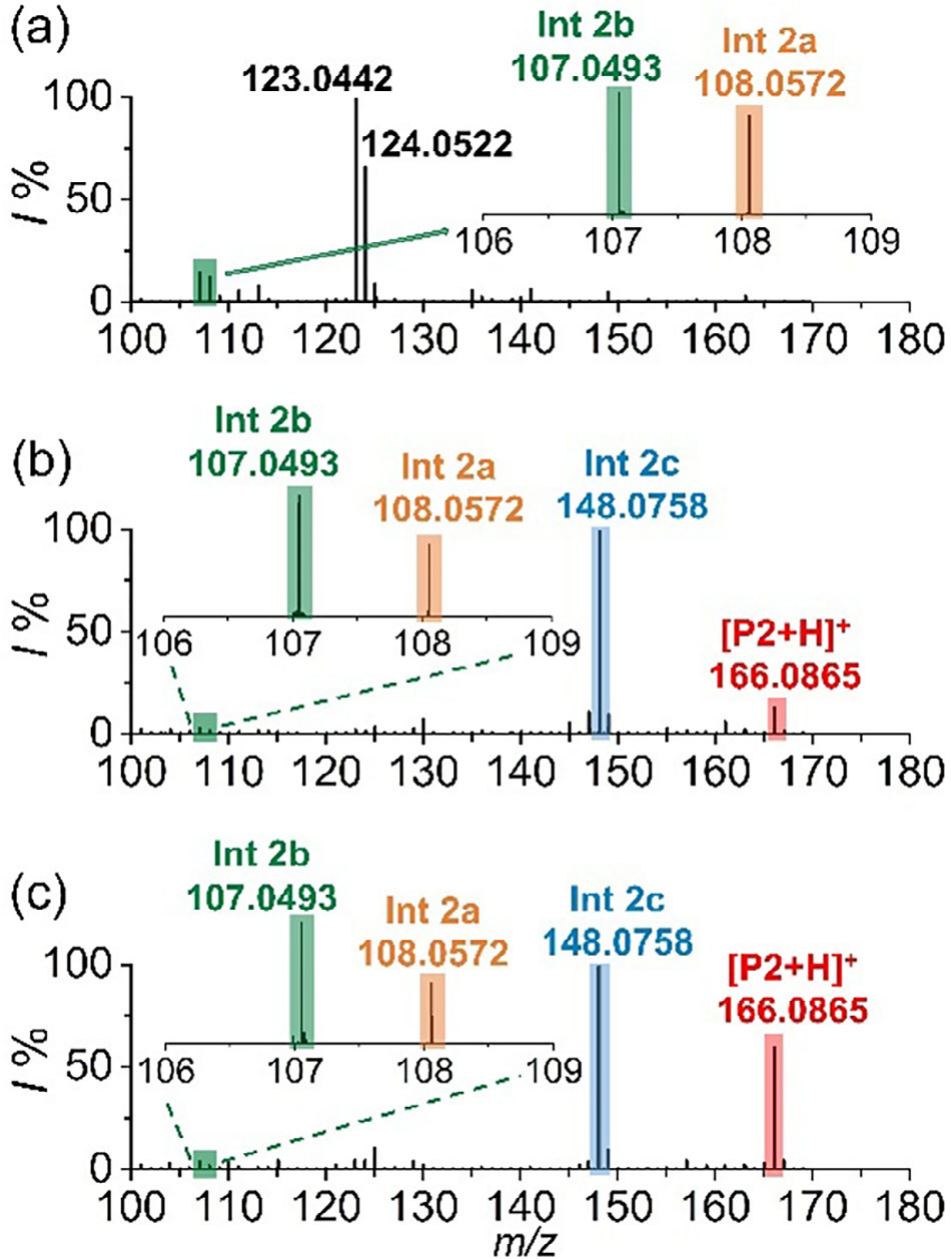
Mass spectra of 2‐methylphenol (1 mm) obtained using a hybrid ultramicroelectrode/ion emitter. a) 2‐methylphenol in DCE/HFIP (50:50), b) in DCE/HFIP/ACN (47.5:47.5:5), and c) in DCE/HFIP/ACN/Water (47:47:5:1).

**Figure** **5**a shows the mass spectra obtained from DCE/HFIP containing 1 mm 2‐methylphenol (**S2**) using our hybrid ultramicroelectrode/ion emitter. The effective oxidation of **S2** was immediately convinced by a signal at *m/z* 108.0572 (i.e., **Int 2a**, theoretical *m/z* 108.0570, *∆m* = 0.2 mDa, generated via the SET electrooxidation of **S2**). Delightfully, the key phenylic carbocation formed via a further 1 e^−^, 1 H^+^ oxidation of the **Int 2a** was also captured, as evidenced by the obvious signal at *m/z* 107.0493 (**Int 2b**, theoretical *m/z* 107.0491, *∆m* = 0.2 mDa). The identification of the **Int 2a** and **Int 2b** was also convinced by the tandem mass spectrometry (Figure S17a,b, Supporting Information). We also noted the dominant signals located at *m/z* 123.0442 and *m/z* 124.0522, which were probably produced by the reaction of **Int 2b** with the trace residual water present in the solvents. This also suggested the ultrahigh reactivity of **Int 2b**. The successful detection of the **Int 2a** and **Int 2b** proved again the superiority of our device in the capture and identification of the electrogenerated elusive intermediates, attributed to the unique design of the hybrid ultramicroelectrode/ion emitter as well as the stabilization effect of microdroplets.

The reactivity of **Int 2b** with nitrile was then tested by adding anhydrous acetonitrile (5%) into the solvent. As exhibited in Figure 5b, apart from the signals referring to **Int 2a** and **Int 2b**, the expected nitrilium ion (i.e., **Int 2c**) was observed with the highest ion intensity at *m/z* 148.0758 ([C_9_H_10_NO]^+^, theoretical *m/z* 148.0757, *∆m* = 0.1 mDa, see also the Figure S17c (Supporting Information) for structural evaluation by MS/MS). To the best of our knowledge, this is the first time that all intermediates involved in this electrochemical amination (i.e., **Int a**, **Int b,** and **Int c**) were unambiguously identified. Notably, signals from the direct nucleophilic addition of water with **Int b** (i.e., *m/z* 123.0442 and *m/z* 124.0522) almost disappeared. This also explained the high selectivity of this electrochemical amination toward unprotected phenols. Additionally, a distinct peak at *m/z* 166.0865 was observed. It was assigned to the hydrolysed final amide product (**P2**, [C_9_H_11_NO_2_]^+^, theoretical *m/z* 166.0863, *∆m* = 0.2 mDa, see Figure S17d (Supporting Information) for structural evaluation by MS/MS), due to the presence of trace residual water in the DCE/HFIP solvent system. The hydrolysis of the **Int 2c** was further rationalized by adding water (1%) into the reaction media, leading to a significant increase in the relative ion intensity of the amide product (see Figure 5b,c for comparison). Despite the presence of a methyl group in **S2**, the off‐line electrosynthesis (∼70% yield) and product analysis using ^1^H NMR (Figure S18, Supporting Information) revealed that the amination of **S2** is highly *para*‐selective, i.e., the C(sp^2^)‐H amination rather than the C(sp^3^)‐H amination. Theoretical analysis using DFT calculations also revealed that the formation of carbocation at the sp3 carbon involves the thermodynamically unfavorable isomerization steps. In particular, the isomerization of **Int 2b** to produce the benzyl carbocation is of a free energy change of ≈30 kcal mol^−1^ (Figure S19, Supporting Information), which is practically “impossible” to proceed at room temperature (≫10 kcal mol^−1^). Accordingly, we concluded that the electrochemically activated site of **Int 2b** is primarily at the *para* position to afford the position‐selective amination via a Ritter‐like mechanism (Figure 4).

In addition, other substituted phenol derivatives were also tested (see Figures S20–S22 and Tables S7 and S8, Supporting Information as well as the Figures S23 and S24, Supporting Information for tandem mass spectrometry), with the successful detection of the electrogenerated involved intermediates as well as the final amination products. The above results thus have collectively demonstrated that, in addition to identification of the electrogenerated key intermediates, valuable information on their ultrafast subsequent transformations can also be stepwisely obtained by in situ mass spectrometry.

## 3 Conclusion

In conclusion, by virtue of the low‐delay implementation of electrooxidation and nanoelectrospray ionization, we achieved the rapid and direct mass spectrometric identification of the electrogenerated benzylic and phenylic carbocation intermediates. The stepwise evaluation of their initial electrochemical generation as well as their ultrafast succedent chemical transformations provided valuable insights into the detailed paths of the carbocation‐mediated C(sp^3^)–H and C(sp^2^)–H aminations. Importantly, the confined fabrication of an ultramicroelectrode into a tapered micropipette permitted the ultrafast transfer of the electrogenerated species into the stabling microdroplets, which is the key to the successful detection of the electrogenerated nanosecond‐lived carbocations. The low‐delay feature of the in situ electrochemical mass spectrometry not only provided a powerful tool for identification of the previously unseen intermediates at a new time window, but also paved the way for in‐depth exploration of the detailed electrochemical processes.

## 4 Experimental Section

### Chemicals and Materials

Hexafluoroisopropanol (HFIP, 99%), 1,2‐Dichloroethane (DCE, 99.5%), 4‐Ethylbiphenyl (**S1**, 99%), Ethylbenzene (99%), p‐Ethyltoluene (98%), 1,3,5‐Triethylbenzene (90%), 1,2,3,4‐Tetrahydronaphthalene (98%), 1‐Chloro‐4‐ethylbenzene (97%), 1‐Ethyl‐4‐Fluorobenzene (98%), 4‐Nitropyrazole (98%), Methyl 4‐pyrazolecarboxylate (98%), 3‐Chloro‐1,2,4‐triazole (97%), 3‐(Trifluoromethyl)‐1H‐1,2,4‐triazole (98%), 3‐Methyl‐1H‐1,2,4‐trizole (98%), 3,5‐Dimethyl‐4H‐1,2,4‐triazole (97%), Methyl 2H‐1,2,4‐triazole‐4‐carboxylate (97%), Pyridine (99.5%), 3‐Nitropyridine (98%), 4‐Methoxypyridine (98%), 4‐(Trifluoromethyl)pyridine (98%), 3‐Chloropyridine (99%), 4‐Cyanopyridine (99%), 4‐Pyridinecarboxaldehyde (98%), 2‐Picoline (99%), 3,5‐Lutidine (99%), 4‐Ethylpyridine (98%), Acetamide (98%), Urea (99%), Purine (98%), Quinoline (99%), Pyridazine (98%), 2‐Methylphenol (**S2**, 99%), 2,6‐Dimethylphenol (99%), 2‐tert‐Butylphenol (99%) and anhydrous Acetonitrile (99.8%) were purchased from Adamas (Shanghai, China). N‐Butylbenzene (99%), 1,4‐Diethylbenzene (99%), p‐Ethylanisol (98%), 4,4′‐Diethylbiphenyl (98%), Diphenylmethane (98%), Hydrindene (95%), Pyrazole (98%), 4‐Chloropyrazole (98%), 4‐Bromopyrazole (99%), 4‐(Trifluoromethyl)‐1H‐pyrazole (97%), 4‐Methylpyrazole (98%), 1,2,4‐Triazole (98%) and Benzylamine (99%) were purchased from Aladdin (Shanghai, China). N‐propylbenzene (98%) was purchased from Macklin (Shanghai, China). Pure water was purchased from Wahaha Company (Hangzhou, China). All chemicals were used without further purification. The borosilicate glass capillaries (i.d. = 0.58 mm, o.d. = 1 mm, with filament) were purchased from Sutter Instruments.

### Fabrication and Characterization of the Hybrid Ultramicroelectrode/Ion Emitter

A tapered ion emitter (≈2 µm opening) was first fabricated using a CO_2_ laser puller (Model P‐2000, Sutter Instrument Co., Novato, CA, USA) from a borosilicate glass capillary. Typical parameters of the laser puller were as follows:

(1) Line1:HEAT:420,FIL:4,VEL:30,DEL:132,PUL:66

As illustrated in Figure S1 (Supporting Information), the hybrid ultramicroelectrode/ion emitter was then fabricated by sequentially decorating a thin layer of chromium (Cr, ≈5 nm in thickness, as an adhesive layer) and platinum (Pt, ≈100 nm in thickness) onto the outer surface of a bare emitter and into the inner surface of its front tip, using a vacuum coating apparatus (GVC‐2000T, Gevee Instrumen Co., Beijing, China). Except as noted, the instrument parameters of chromium decoration were as follows: time = 6 s, current = 160 mA; the instrument parameters of platinum decoration were as follows: time = 112 s, current = 120 mA. The overall thickness of the deposited Pt is ≈100 nm.

A photograph of three bare ion emitters and three hybrid ultramicroelectrodes/ion emitters were presented in Figure S2a (Supporting Information). Optical images in Figure S2b (Supporting Information) was recorded with an Olympus IX73 microscope. Figure S2a,b (Supporting Information) clearly demonstrated the morphological changes of the ion emitter after platinum sputtering. The hybrid ultramicroelectrode/ion emitter exhibited an opaque appearance with a metallic platinum luster. A scanning electron microscope (SEM, SM‐7800F, JEOL Ltd., Japan) was also used for characterizing the hollow structure of the hybrid ultramicroelectrode. The tip diameter was measured to be ≈2 µm (Figure 1d). To characterize the Pt deposit inside the tip, focused ion beam cutting was also performed using the Helios600i dual‐beam electron microscopic system (FEI, Hillsboro, OR, U.S.A.). According to the high‐resolution SEM image of the sample section, the length of the platinum deposit inside the emitter tip was ≈5 µm (Figure 1e). These results collectively confirmed the successful fabrication of the Pt ultramicroelectrode into the front tip of an ion emitter.

### Mass Spectrometry

All mass spectra were acquired using the LTQ‐Orbitrap hybrid mass spectrometer (Velos, Thermo Fisher Scientific Co., Waltham, Massachusetts, U.S.A.). The ultramicroelectrode was placed in front of the MS inlet at a distance of ≈5 mm. Except as noted, the instrument parameters were as follows: positive ion mode, spray voltage = 0.8–1.6 kV, capillary temperature = 350 °C, maximum injection time = 50 ms, resolution = 30 000. Isotopic Distribution Calculator (Release B633.0, Agilent) was used for calculating the theoretical isotopic distributions.

### Off‐Line Electrosynthesis

The off‐line electrosynthesis were carried out using an undivided cell with the optimized reaction conditions^[^ ^45^, ^46^ ^]^ (graphite anode, platinum cathode, *o*‐Cresol (0.5 mmol, 0.1 M), Bu_4_NPF_6_ (2 equiv), CH_3_CN (4 mL), constant current = 6.0 mA, 6 h (3.7 F mol^−1^), room temperature), which afforded N‐(4‐hydroxy‐3‐methylphenyl)acetamide with a purified yield of ≈70%.

## Conflict of Interest

The authors declare no conflict of interest.

## Supporting information



Supporting Information

## Data Availability

The data that support the findings of this study are available from the corresponding author upon reasonable request.

## References

[1] C. Kingston , M. D. Palkowitz , Y. Takahira , J. C. Vantourout , B. K. Peters , Y. Kawamata , P. S. Baran , Acc. Chem. Res. 2019, 53, 72. 31823612 10.1021/acs.accounts.9b00539 PMC6996934

[2] P. Xiong , H.‐C. Xu , Acc. Chem. Res. 2019, 52, 3339. 31774646 10.1021/acs.accounts.9b00472

[3] Z. Yang , W. Shi , H. Alhumade , H. Yi , A. Lei , Nat. Synth. 2023, 2, 217.

[4] Y. Yuan , A. Lei , Acc. Chem. Res. 2019, 52, 3309. 31774271 10.1021/acs.accounts.9b00512

[5] L. Zeng , Q. Yang , J. Wang , X. Wang , P. Wang , S. Wang , S. Lv , S. Muhammad , Y. Liu , H. Yi , Science 2024, 385, 216. 38991063 10.1126/science.ado0875

[6] L. Zeng , J. Wang , D. Wang , H. Yi , A. Lei , Angew. Chem., Int. Ed. 2023, 62, 202309620. 10.1002/anie.202309620 37606535

[7] L. Zeng , Y. Jiao , W. Yan , Y. Wu , S. Wang , P. Wang , D. Wang , Q. Yang , J. Wang , H. Zhang , Nat. Synth. 2023, 2, 172.

[8] Y. Yu , X.‐B. Zhu , Y. Yuan , K.‐Y. Ye , Chem. Sci. 2022, 13, 13851. 36544744 10.1039/d2sc05423j PMC9710211

[9] Z. W. Hou , D. J. Liu , P. Xiong , X. L. Lai , J. Song , H. C. Xu , Angew. Chem., Int. Ed. 2021, 60, 2943. 10.1002/anie.202013478 33078880

[10] B. C. Hawkins , J. M. Chalker , M. L. Coote , A. C. Bissember , Angew. Chem. 2024, 136, 202407207. 10.1002/anie.202407207 39075778

[11] Q. Lin , Y. Duan , Y. Li , R. Jian , K. Yang , Z. Jia , Y. Xia , L. Zhang , S. Luo , Nat. Commun. 2024, 15, 6900. 39134515 10.1038/s41467-024-50945-2 PMC11319787

[12] M. Takumi , H. Sakaue , A. Nagaki , Angew. Chem. 2022, 134, 202116177. 10.1002/anie.202116177 34931424

[13] W. Zeng , C. Peng , Y. Qiu , J. Am. Chem. Soc. 2025, 147, 13461. 40203205 10.1021/jacs.5c00259

[14] S. Suga , S. Suzuki , A. Yamamoto , J. Yoshida , J. Am. Chem. Soc. 2000, 122, 10244.

[15] S. Suga , M. Okajima , K. Fujiwara , J. Yoshida , J. Am. Chem. Soc. 2001, 123, 7941. 11493082 10.1021/ja015823i

[16] P. J. Hanway , J. Xue , U. Bhattacharjee , M. J. Milot , Z. Ruixue , D. L. Phillips , A. H. Winter , J. Am. Chem. Soc. 2013, 135, 9078. 23713909 10.1021/ja403370k

[17] S. Jaarinen , J. Niiranen , J. Koskikallio , Int. J. Chem. Kinet. 1985, 17, 925.

[18] H. Meerwein , K. van Emster , Ber. Dtsch. Chem. Ges. (A and B Series) 1922, 55, 2500.

[19] G. A. Olah , Carbocation Chem. 2004, 7.

[20] G. S. Prakash , Curr. Sci. 1995, 68, 14.

[21] W. Chen , X. Yi , Z. Liu , X. Tang , A. Zheng , Chem. Soc. Rev. 2022, 51, 4337. 35536126 10.1039/d1cs00966d

[22] V. Mišić , K. Piech , T. Bally , J. Am. Chem. Soc. 2013, 135, 8625. 23621766 10.1021/ja401890m

[23] F. Braak , H. Elferink , K. J. Houthuijs , J. Oomens , J. Martens , T. J. Boltje , Acc. Chem. Res. 2022, 55, 1669. 35616920 10.1021/acs.accounts.2c00040 PMC9219114

[24] P. Das , Chem. Rev. 1993, 93, 119.

[25] T. A. Brown , H. Chen , R. N. Zare , J. Am. Chem. Soc. 2015, 137, 7274. 26030136 10.1021/jacs.5b03862

[26] T. A. Brown , H. Chen , R. N. Zare , Angew. Chem. 2015, 127, 11335. 10.1002/anie.201506316 26352029

[27] T. A. Brown , N. Hosseini‐Nassab , H. Chen , R. N. Zare , Chem. Sci. 2016, 7, 329. 28791096 10.1039/c5sc02939b PMC5518571

[28] J. Hu , T. Wang , W. J. Zhang , H. Hao , Q. Yu , H. Gao , N. Zhang , Y. Chen , X. H. Xia , H. Y. Chen , Angew. Chem., Int. Ed. 2021, 60, 18494. 10.1002/anie.202106945 34129259

[29] J. Hu , N. Zhang , P. K. Zhang , Y. Chen , X. H. Xia , H. Y. Chen , J. J. Xu , Angew. Chem. 2020, 132, 18401.

[30] J. Chen , X. Wang , X. Cui , Y. Li , Y. Feng , Z. Wei , Angew. Chem. 2023, 135, 202219302. 10.1002/anie.202219302 36710258

[31] X. Liu , J. Chen , Z. Wei , H. Yi , A. Lei , Chem 2024, 10, 2131.

[32] A. Kumar , S. Mondal , S. Banerjee , J. Am. Chem. Soc. 2021, 143, 2459. 33534557 10.1021/jacs.0c12512

[33] A. Kumar , S. Mondal , P. V. Sandeep , A. Kumar , S. Banerjee , J. Am. Chem. Soc. 2022, 144, 3347. 35179907 10.1021/jacs.1c12644

[34] A. Kumar , S. Mondal , M. Mofidfar , R. N. Zare , S. Banerjee , J. Am. Chem. Soc. 2022, 144, 7573. 35452233 10.1021/jacs.2c01577

[35] C.‐Y. Liu , Y. Chen , J. Hu , Anal. Chem. 2024, 96, 3354. 38295431 10.1021/acs.analchem.3c04315

[36] H. Cheng , T. Yang , M. Edwards , S. Tang , S. Xu , X. Yan , J. Am. Chem. Soc. 2022, 144, 1306. 35015550 10.1021/jacs.1c11179

[37] S. Tang , H. Cheng , X. Yan , Angew. Chem., Int. Ed. 2020, 59, 209. 10.1002/anie.201911070 31639243

[38] G. J. Van Berkel , F. Zhou , Anal. Chem. 1995, 67, 2916.

[39] G. J. Van Berkel , F. Zhou , Anal. Chem. 1995, 67, 3958.

[40] Q. Wan , S. Chen , A. K. Badu‐Tawiah , Chem. Sci. 2018, 9, 5724. 30079181 10.1039/c8sc00251g PMC6050606

[41] G. J. Van Berkel , K. G. Asano , P. D. Schnier , J. Am. Soc. Mass Spectrom. 2001, 12, 853. 11444609 10.1016/S1044-0305(01)00264-1

[42] A. S. Siegel , J. Am. Chem. Soc. 1970, 92, 5277.

[43] L. Shi , R. A. LaCour , N. Qian , J. P. Heindel , X. Lang , R. Zhao , T. Head‐Gordon , W. Min , Nature 2025, 640, 87. 40108466 10.1038/s41586-025-08702-y PMC12335212

[44] H. Hao , I. Leven , T. Head‐Gordon , Nat. Commun. 2022, 13, 280. 35022410 10.1038/s41467-021-27941-x PMC8755715

[45] C.‐Y. Hsu , G. R. D. Prabhu , C.‐H. Chang , P.‐C. Hsu , K. Buchowiecki , P. L. Urban , Anal. Chem. 2023, 95, 14702. 37725015 10.1021/acs.analchem.3c02799

[46] D. N. Mortensen , E. R. Williams , J. Am. Chem. Soc. 2016, 138, 3453. 26902747 10.1021/jacs.5b13081

[47] I. M. Taily , D. Saha , P. Banerjee , Org. Lett. 2022, 24, 2310. 35312329 10.1021/acs.orglett.2c00439

