# Supplementary material for: Microdroplet Stabilization Enabled Direct Mass Spectrometric Identification of Electrogenerated Transient Carbocation Intermediates
Source: Adv Sci (Weinh). 2025 Dec 8;13(9):e18157. doi: 10.1002/advs.202518157 (PMC12904013; doi:10.1002/advs.202518157)
Supplement: Supplementary file 1 — Supporting Information [file ADVS-13-e18157-s001.pdf]

Supporting Information

Microdroplet Stabilization Enabled Direct Mass Spectrometric Identification of Electrogenerated Transient Carbocation Intermediates

*Guo-Shan Zhu, Ren-Jie Hui and Jun Hu**

G.S. Zhu, R.J. Hui, J. Hu

School of Life Sciences and Health Engineering, Jiangnan University, Wuxi 214122 (China)

E-mail: hujun@jiangnan.edu.cn (J. Hu)

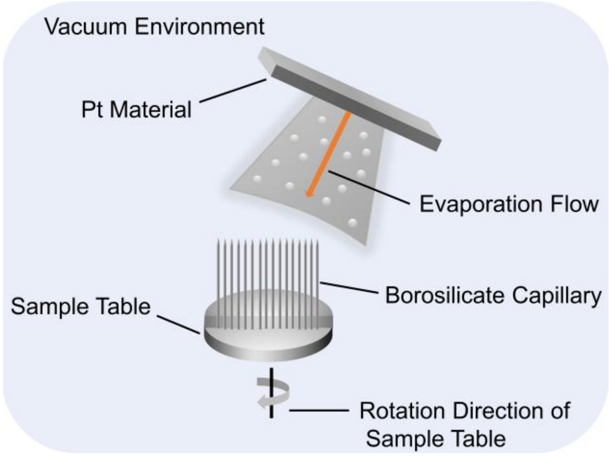

Figure S1. Schematic illustration of the platinum sputtering process.

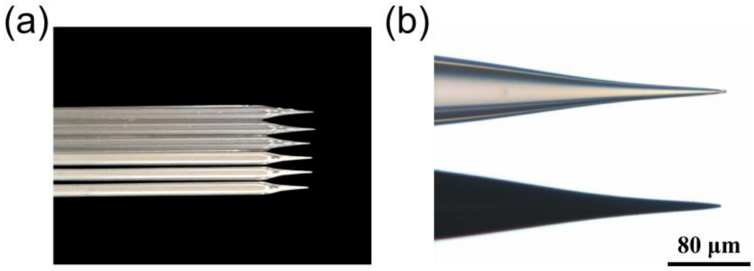

Figure S2. Optical characterization of the ultramicroelectrode/ion emitter. (a) a photograph of three bare (top) and three Pt-decorated (bottom) ion emitters; (b) brightfield microscopic images of a bare (top) and Pt-decorated (bottom) ion emitter.

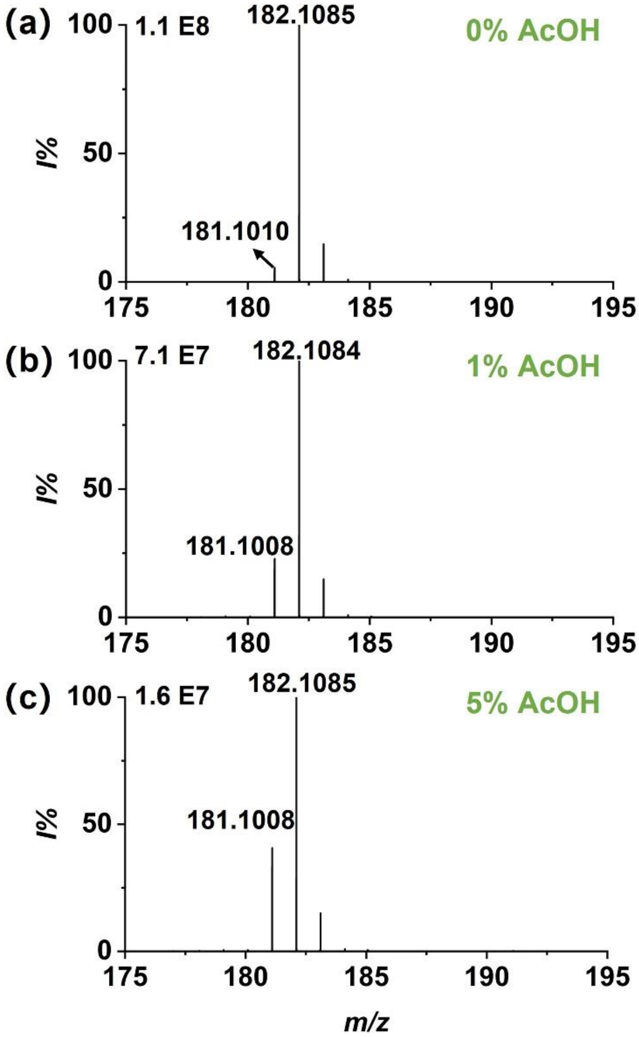

Figure S3. Mass spectra of 4-ethylbiphenyl obtained using a hybrid Pt ultramicroelectrode/ion emitter from DCE/HFIP containing (a) 0% AcOH, (b) 1% AcOH, and (c) 5% AcOH.

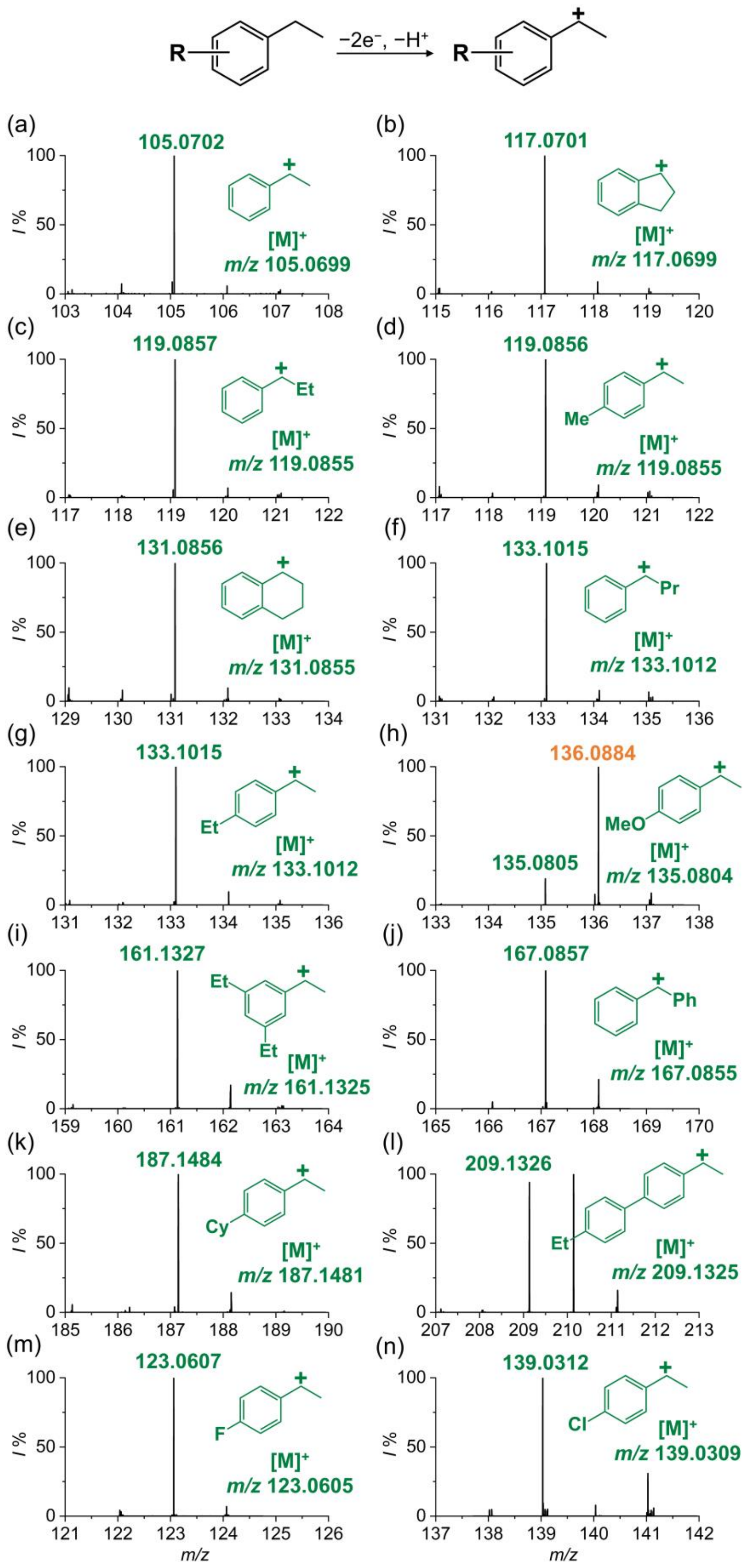

Figure S4. Mass spectra of the electrogenerated carbocations from 14 benzyl derivatives.

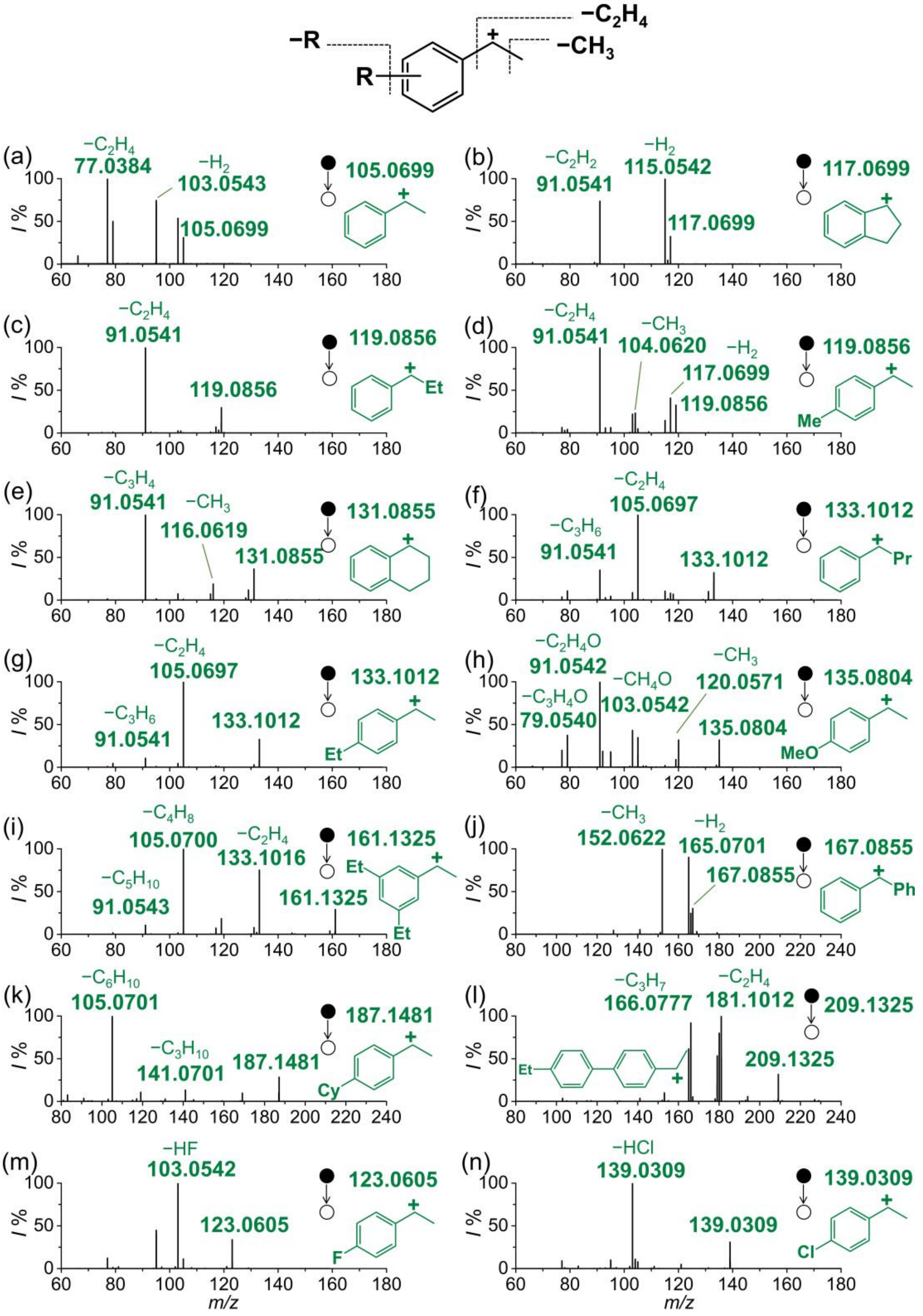

Figure S5. Tandem mass spectrometry of the electrogenerated benzyl carbocations from 14 benzyl derivatives.

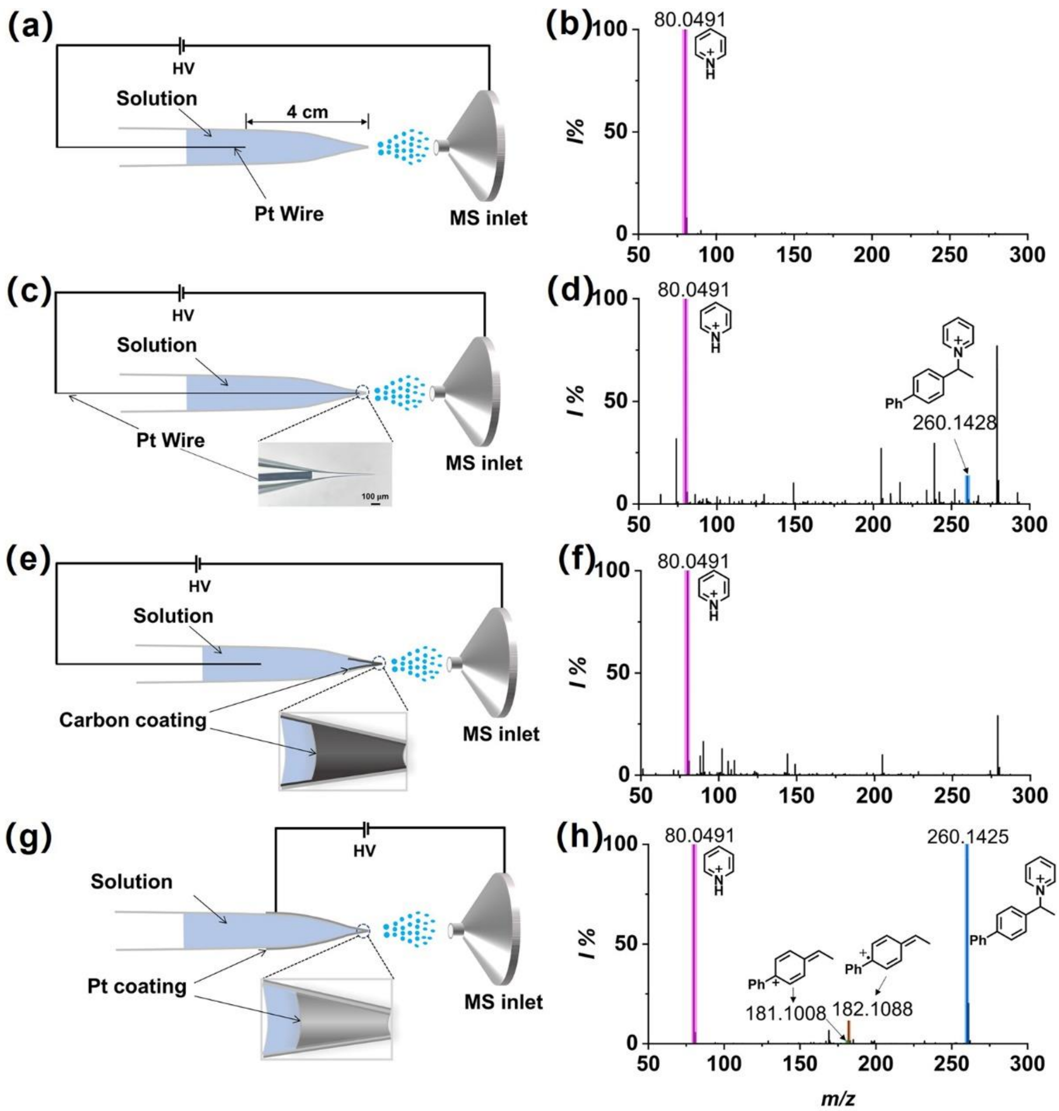

Figure S6. Comparison of the three operation modes: (a) the conventional wire-in-a-capillary approach with the Pt electrode placed >4 cm away from the front tip and (b) the corresponding mass spectrum; (c) the conventional wire-in-a-capillary approach with the Pt electrode inserted into the very front of the tapered tip and (d) the corresponding mass spectrum; (e) our previous BPE-based approach and (f) the corresponding mass spectrum; (g) method in this work and (h) the corresponding mass spectrum. Note only signals referring to the starting substrate (*m/z* 80.0491) and the final product (*m/z* 260.1425) were observed in **Figure S6d**, while no signal referring to the intermediates (**Int 1a** and **Int 1b**) was captured in the conventional wire-in-a-capillary method. Also note the absence of the reaction intermediates and final products in **Figure S6f**, due to the insufficient induced potential on the BPE to activate the electrooxidation of ethyl biphenyl.

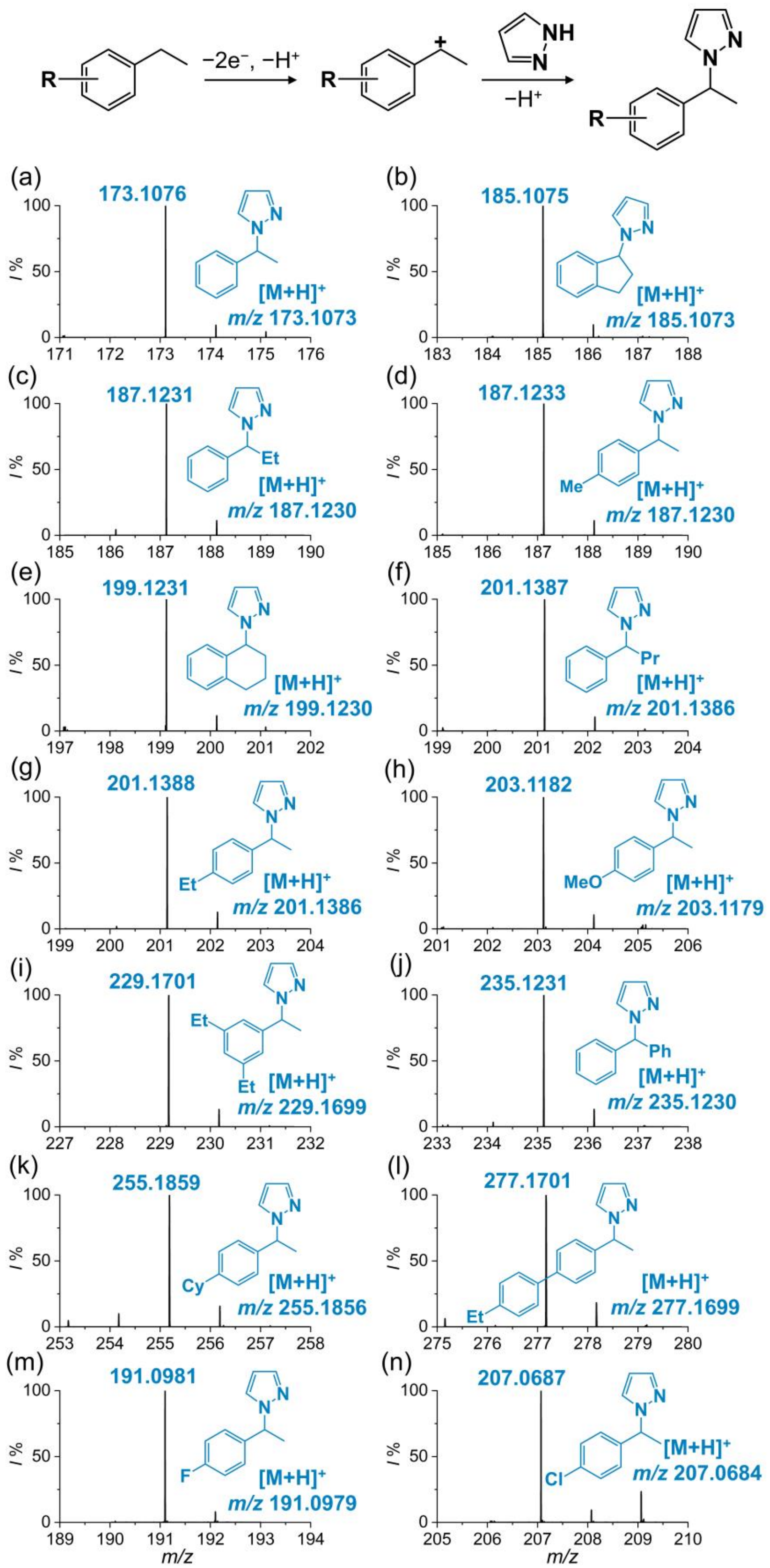

Figure S7. Mass spectra showing the products formed through the nucleophilic addiction of electrogenerated benzyl carbocations by pyrazole.

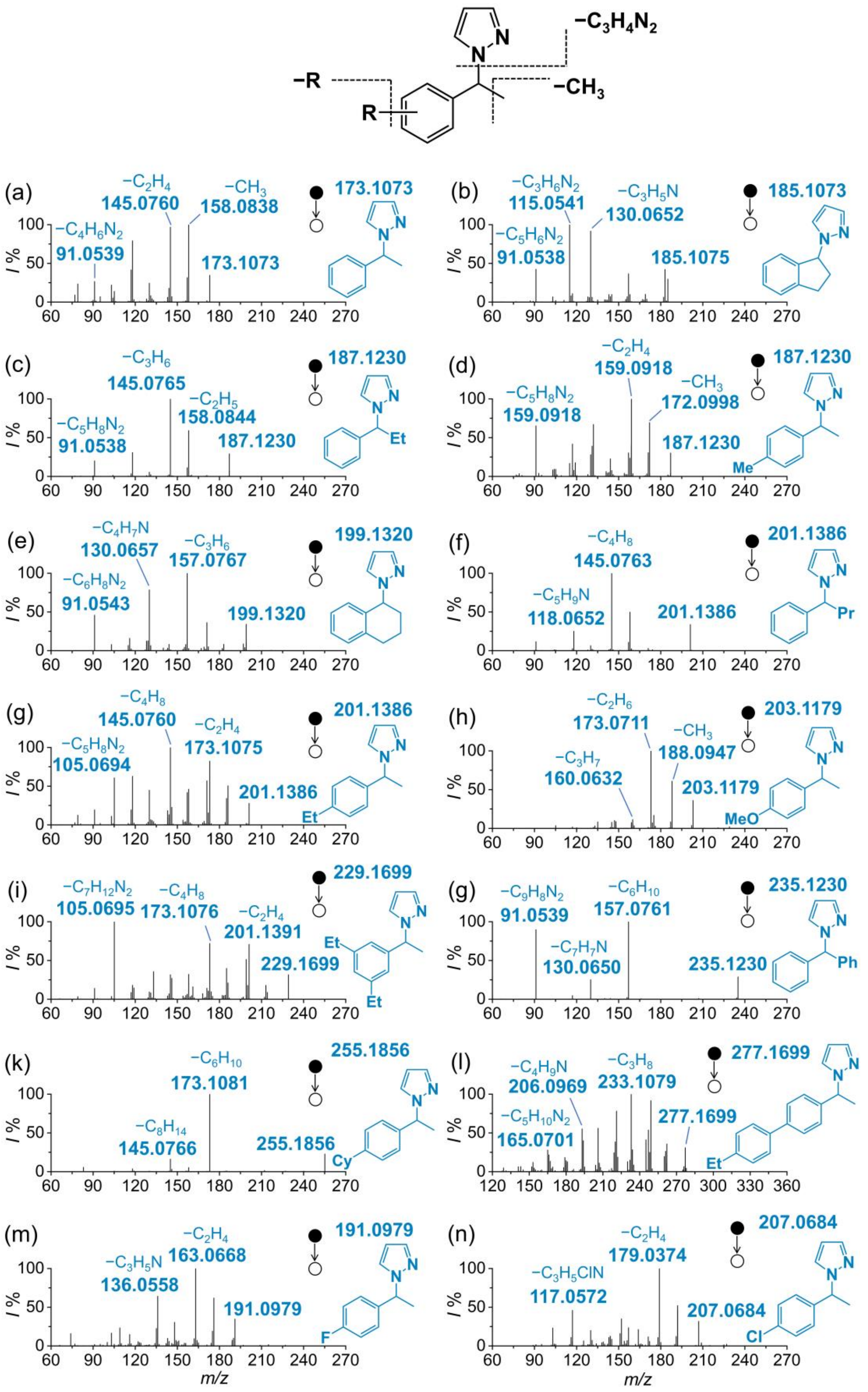

Figure S8. Tandem mass spectrometry of products referring to the pyrazole-mediated C(sp^3)–H aminations.

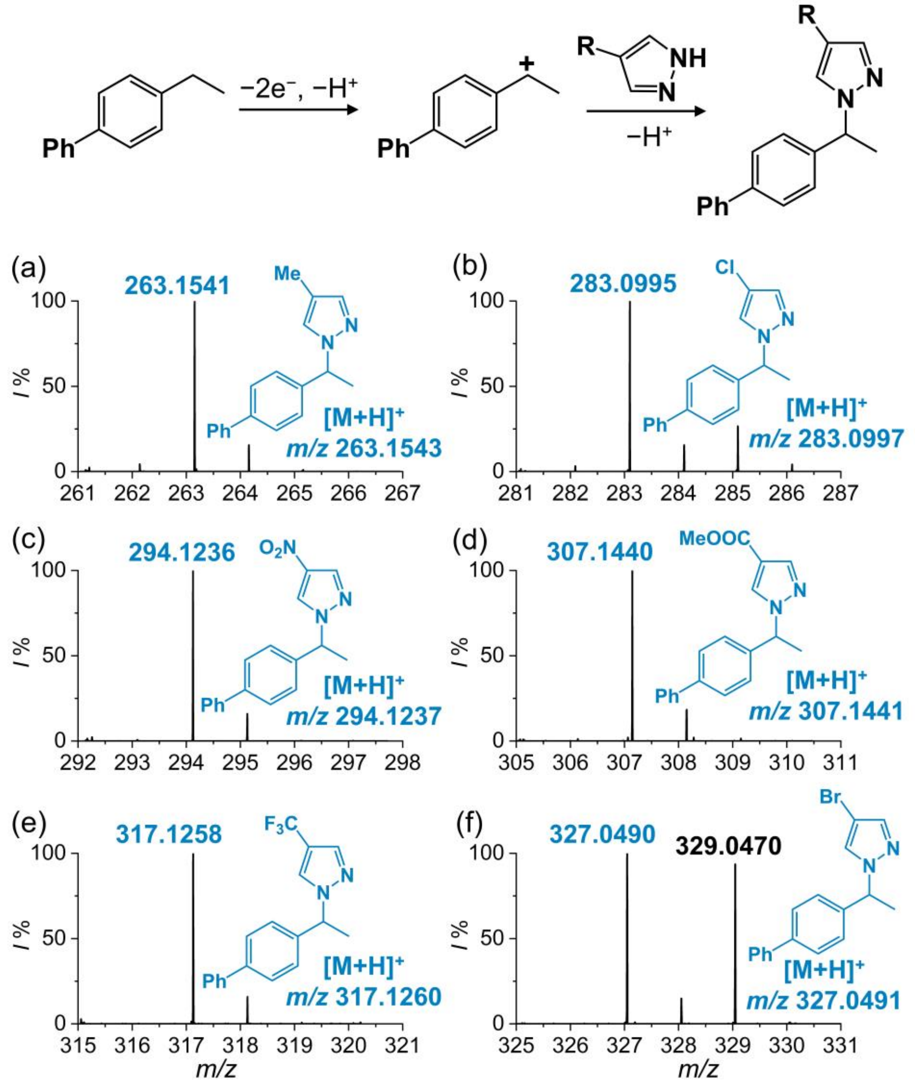

Figure S9. Mass spectra showing the products between the electrogenerated **Int 1b** and the pyrazole derivatives.

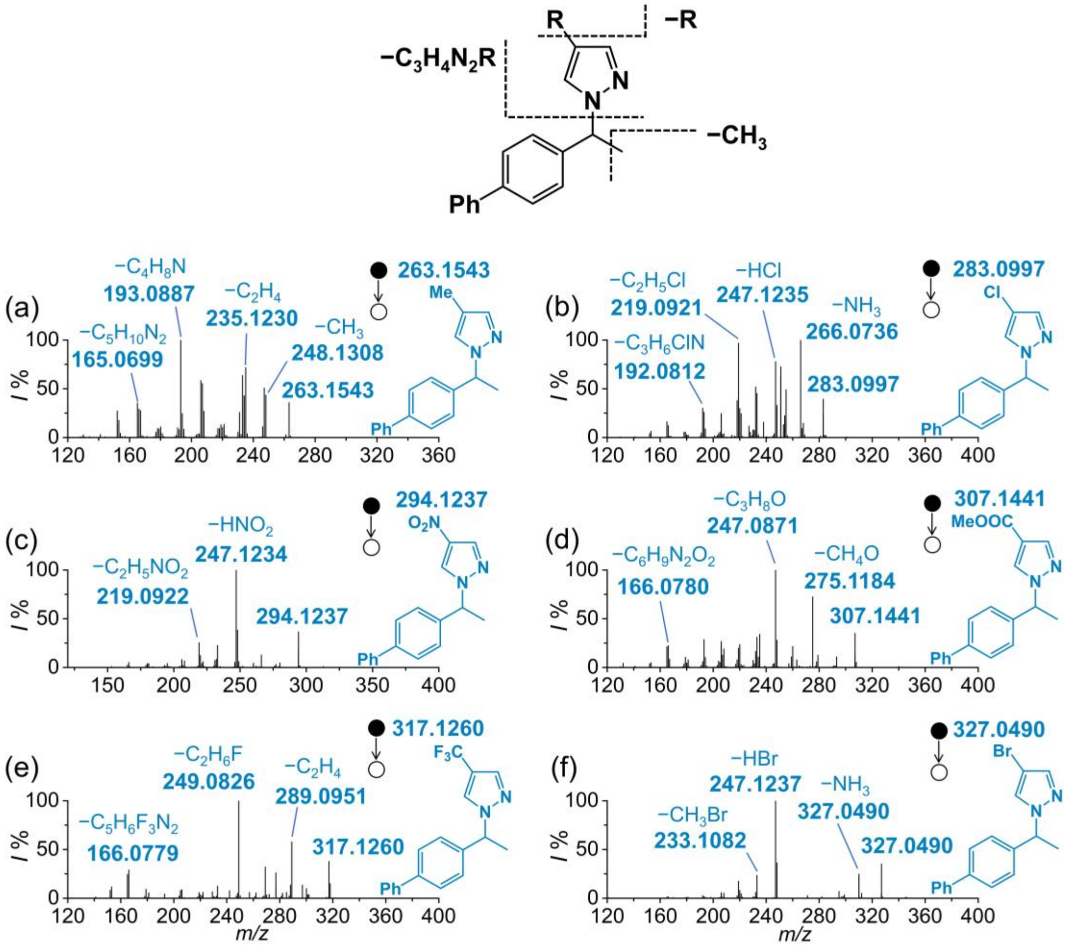

Figure S10. Tandem mass spectrometry of products referring to the pyrazole derivatives mediated C(sp^3)–H aminations.

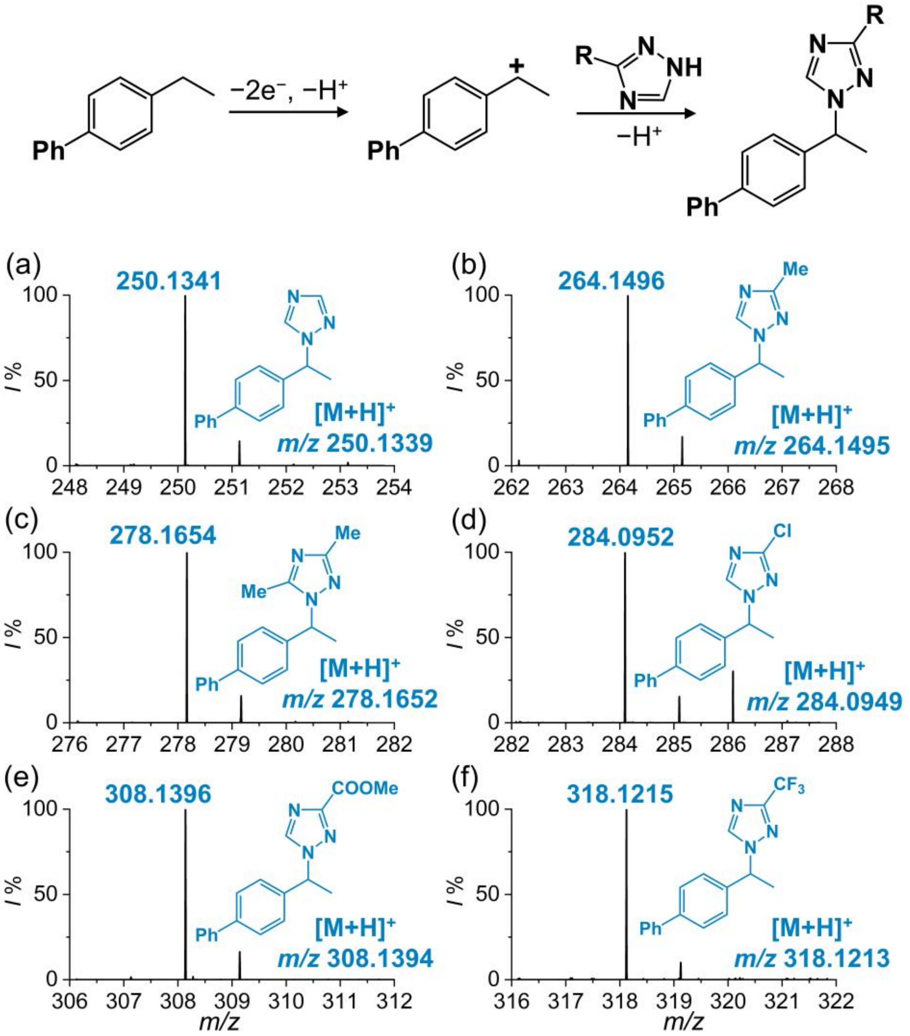

Figure S11. Mass spectra showing the products between the electrogenerated **Int 1b** and the triazole derivatives.

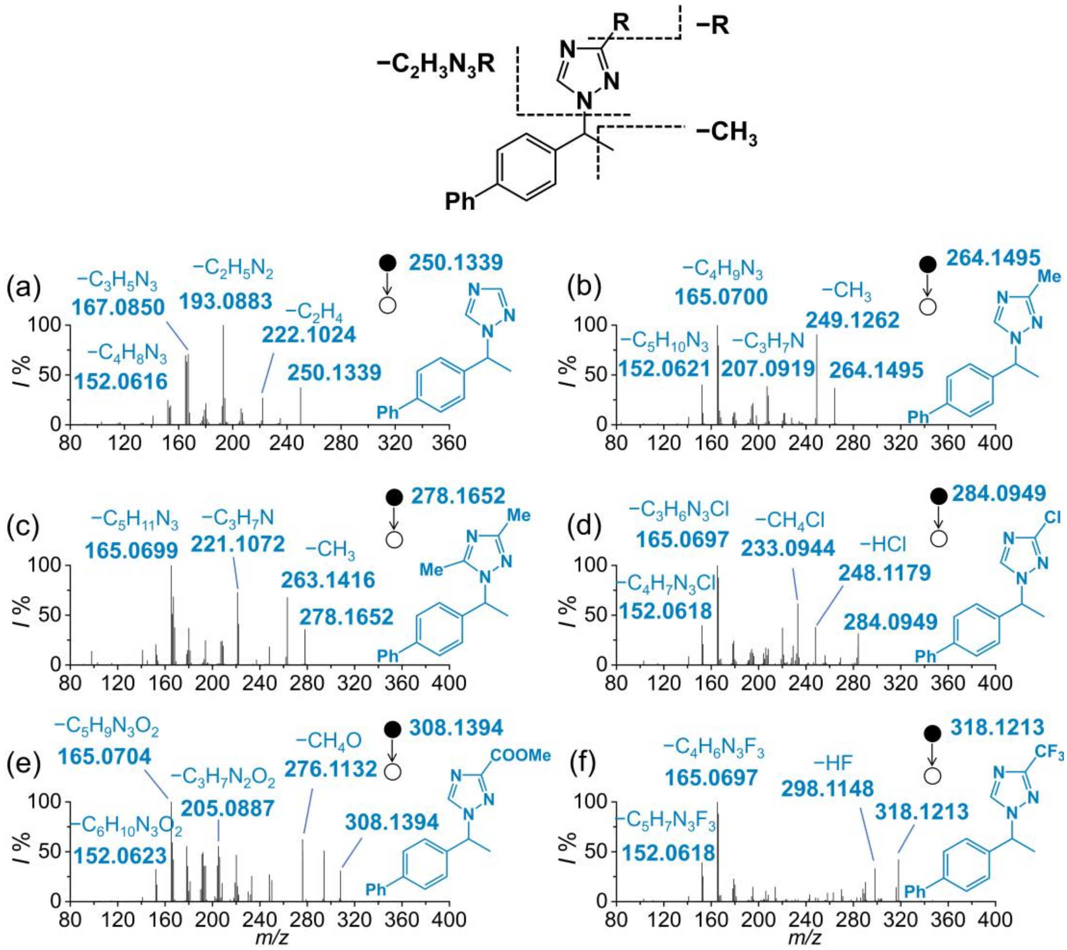

Figure S12. Tandem mass spectrometry of products referring to the triazole derivative mediated C(sp^3)–H aminations.

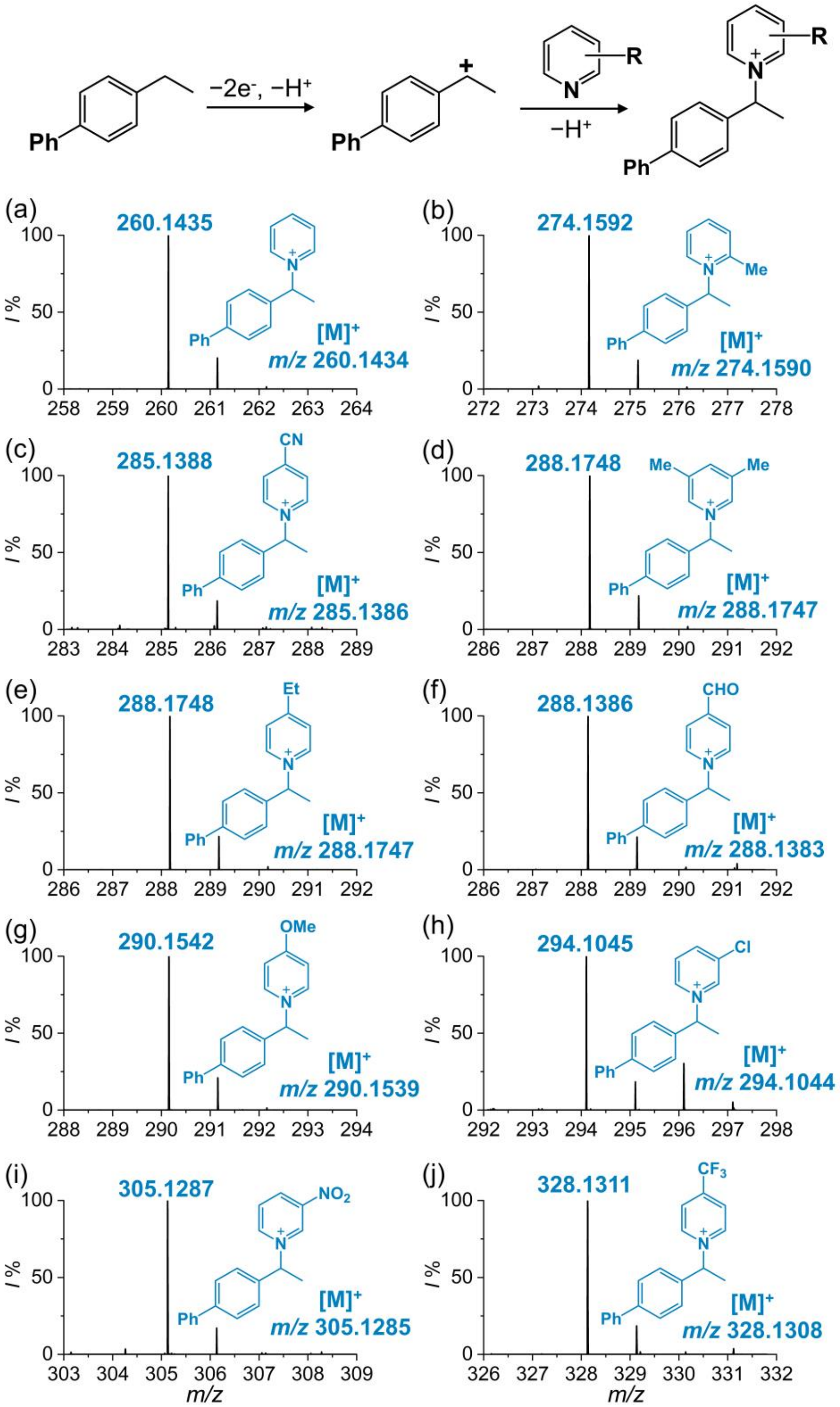

Figure S13. Mass spectra showing the products between the electrogenerated **Int 1b** and the pyridine derivatives.

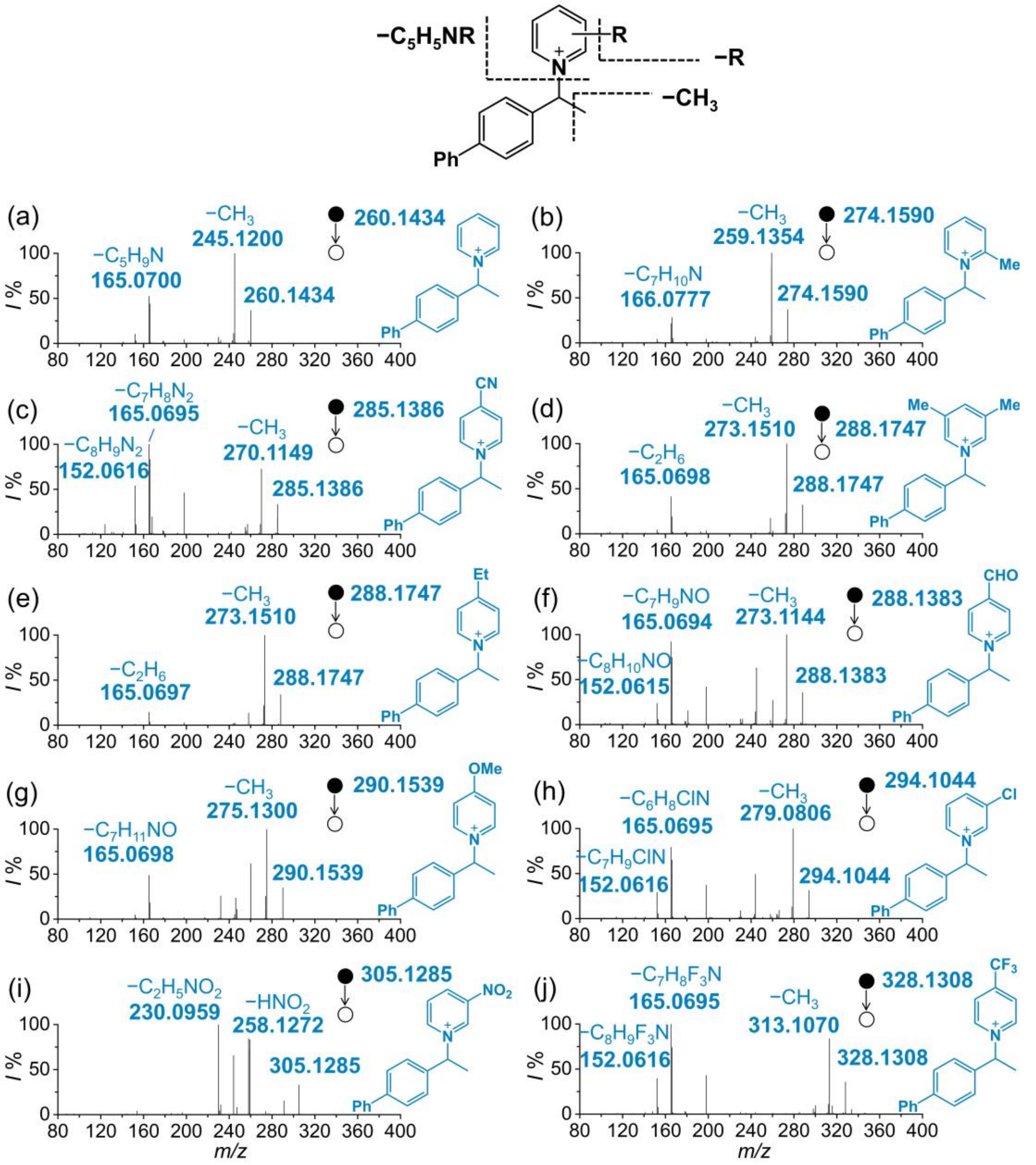

Figure S14. Tandem mass spectrometry of products referring to the pyridine derivative mediated C(sp^3)–H aminations.

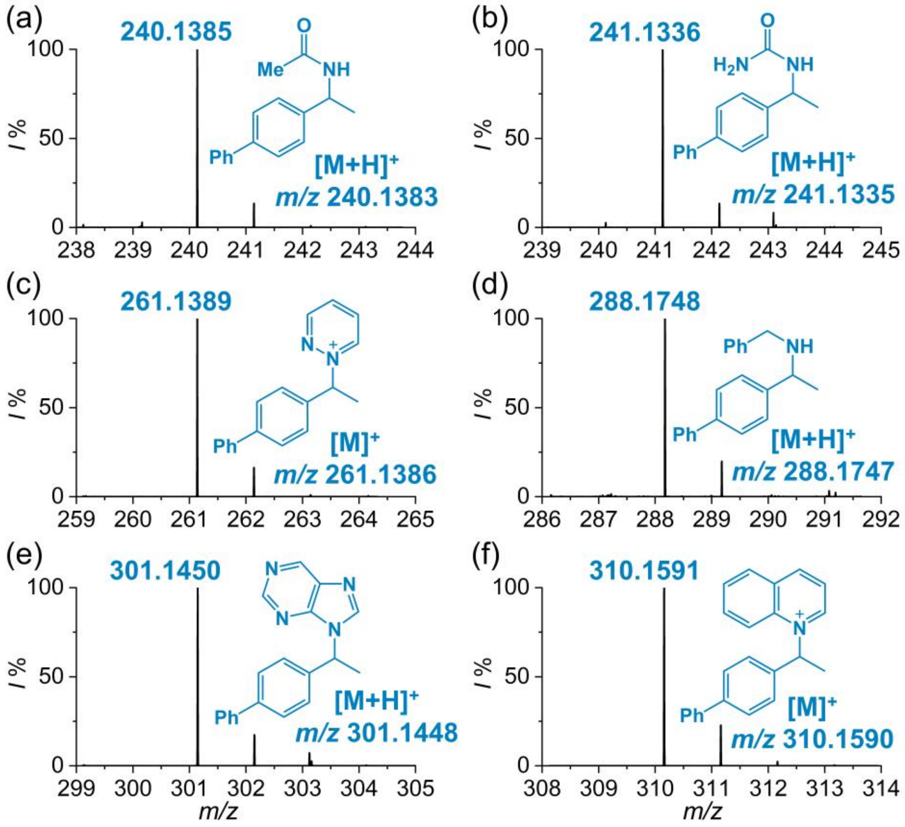

Figure S15. Mass spectra showing the products between the electrogenerated **Int 1b** and the miscellaneous nucleophiles.

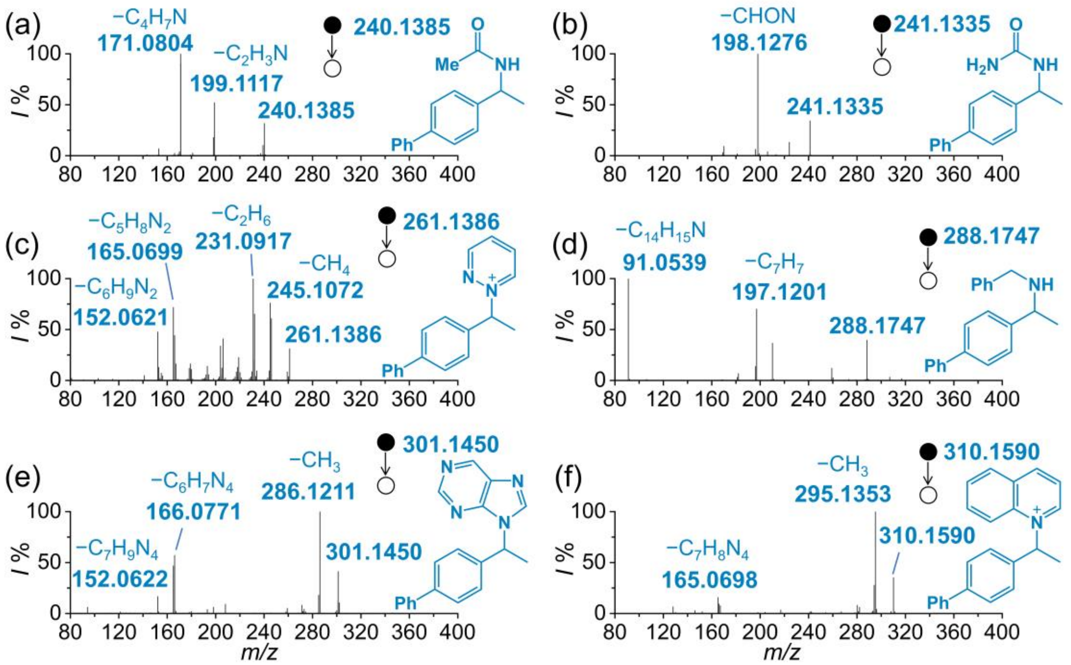

Figure S16. Tandem mass spectrometry of products between the electrogenerated **Int 1b** and the miscellaneous nucleophiles.

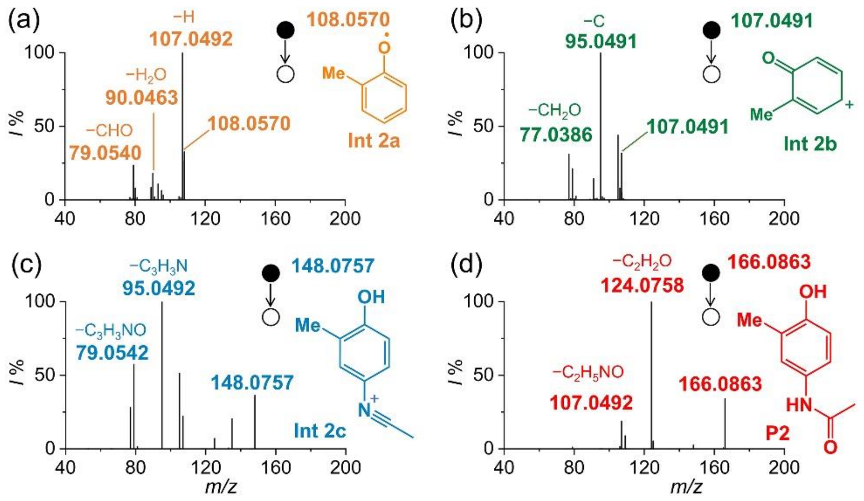

Figure S17. Tandem mass spectrometry of the electrogenerated species upon the anodic oxidation of S2: (a) **Int 2a**, (b) **Int 2b**, (c) **Int 2c** and (d) the final product **P2**.

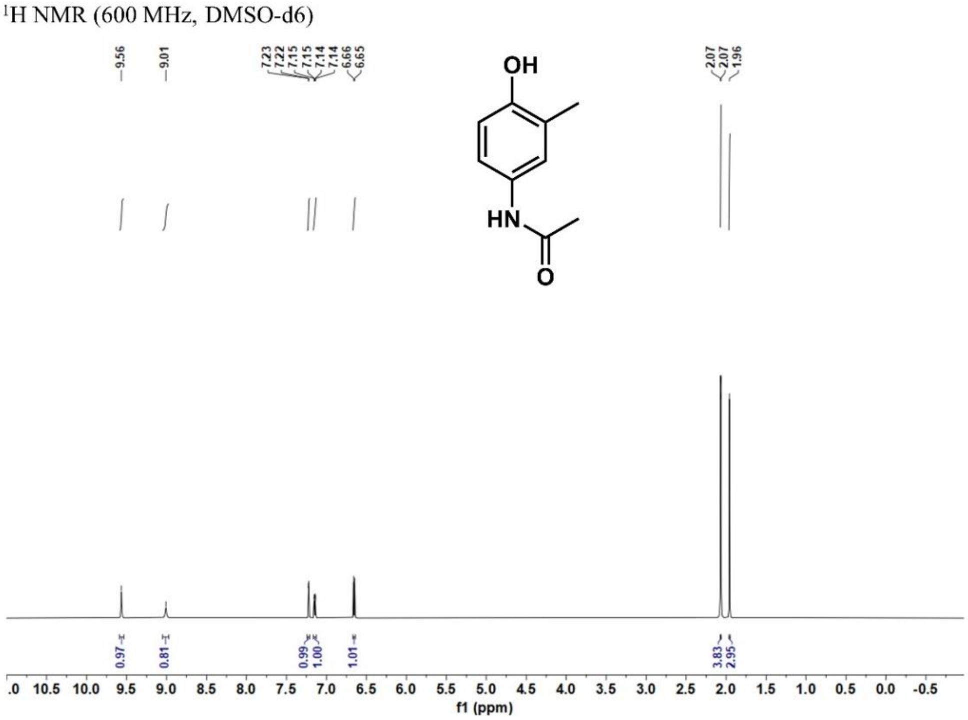

Figure S18. ^{1}H NMR of the chromatography purified product. N-(4-hydroxy-3-methylphenyl)acetamide: (600 MHz, DMSO-d6) δ 9.56 (s, 1H), 9.01 (s, 1H), 7.22 (d, *J* = 2.5 Hz, 1H), 7.15 (dd, *J* = 8.5, 2.6 Hz, 1H), 6.65 (d, *J* = 8.5 Hz, 1H), 2.07 – 2.06 (m, 3H), 1.96 (s, 3H).

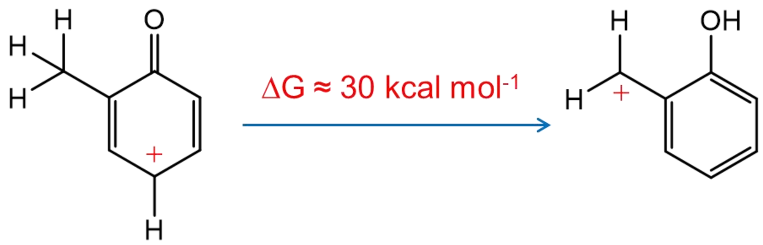

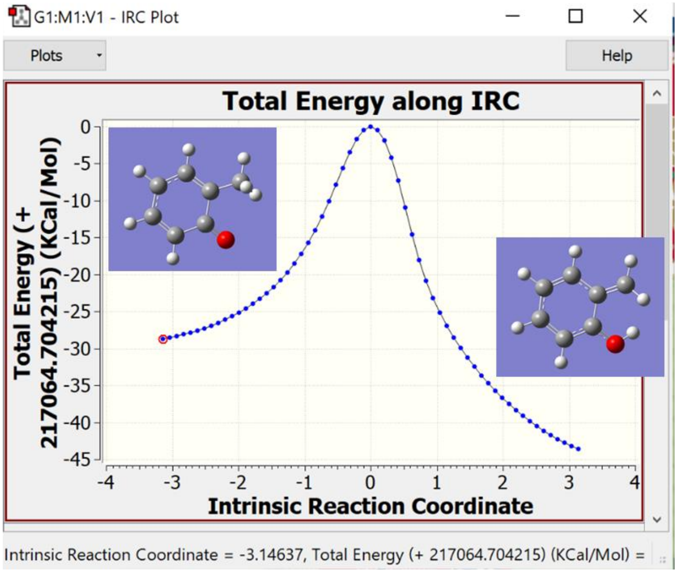

Figure S19. Free energy change for the isomerization of **Int 2b** to produce the benzyl carbocation.

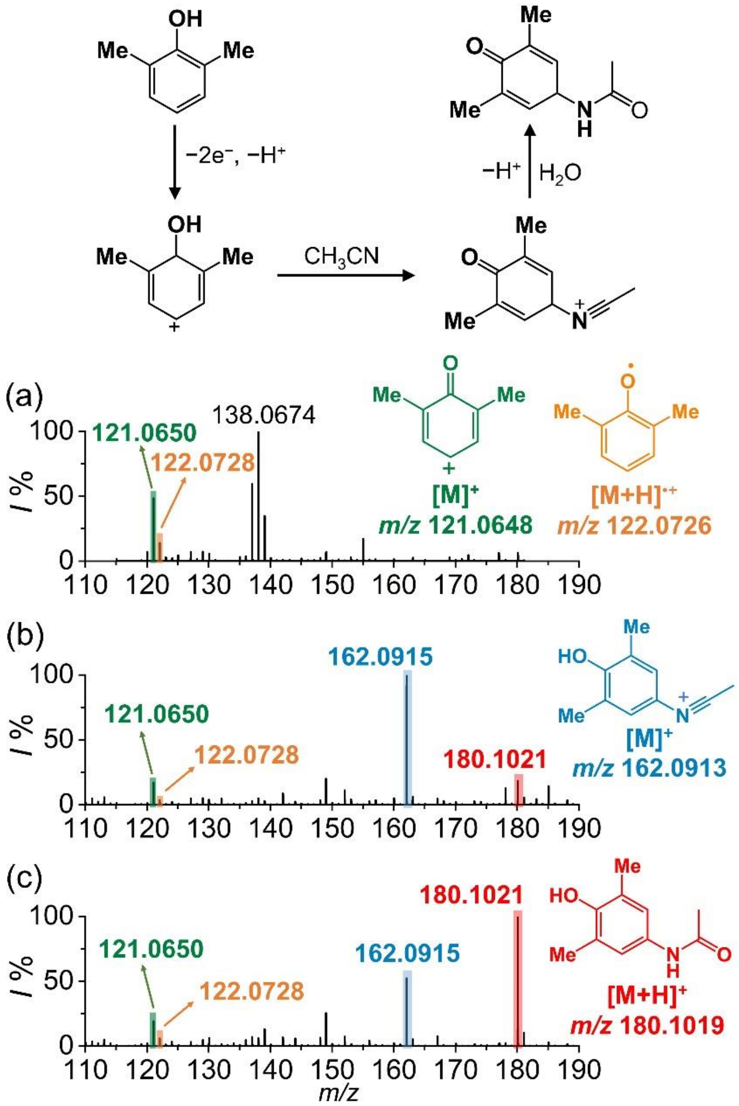

Figure S20. Mass spectra of 2,6-dimethylphenol (1 mM) obtained using a hybrid ultramicroelectrode/ion emitter. (a) 2-methylphenol in DCE/HFIP (50:50), (b) in DCE/HFIP/ACN (47.5:47.5:5), and (c) in DCE/HFIP/ACN/Water (47:47:5:1).

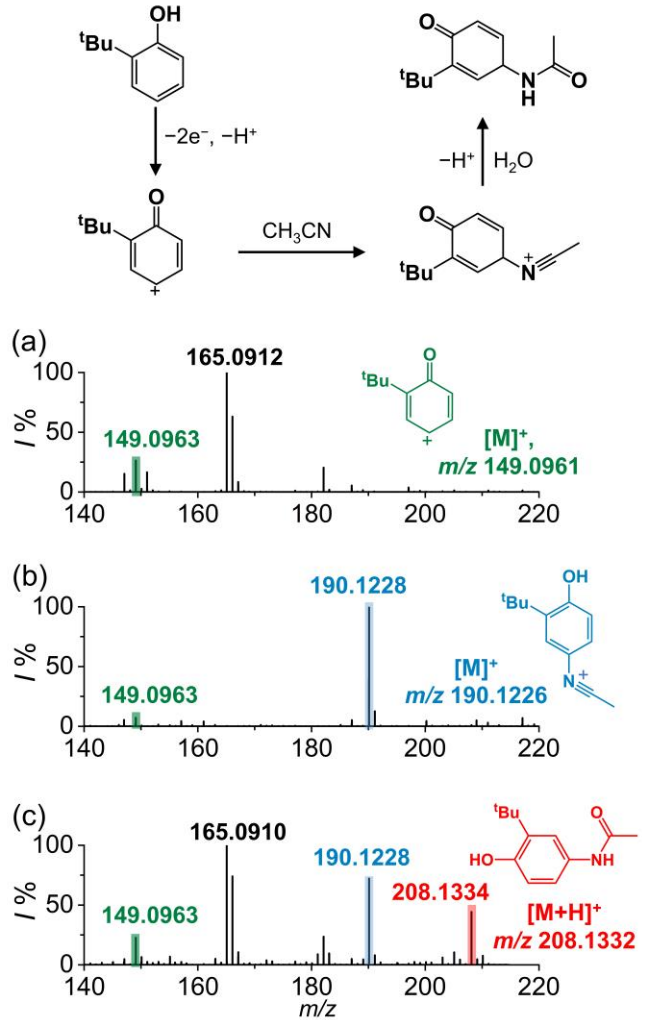

Figure S21. Mass spectra of 2-tert-butylphenol (1 mM) obtained using a hybrid ultramicroelectrode/ion emitter. (a) 2-methylphenol in DCE/HFIP (50:50), (b) in DCE/HFIP/ACN (47.5:47.5:5), and (c) in DCE/HFIP/ACN/Water (47:47:5:1).

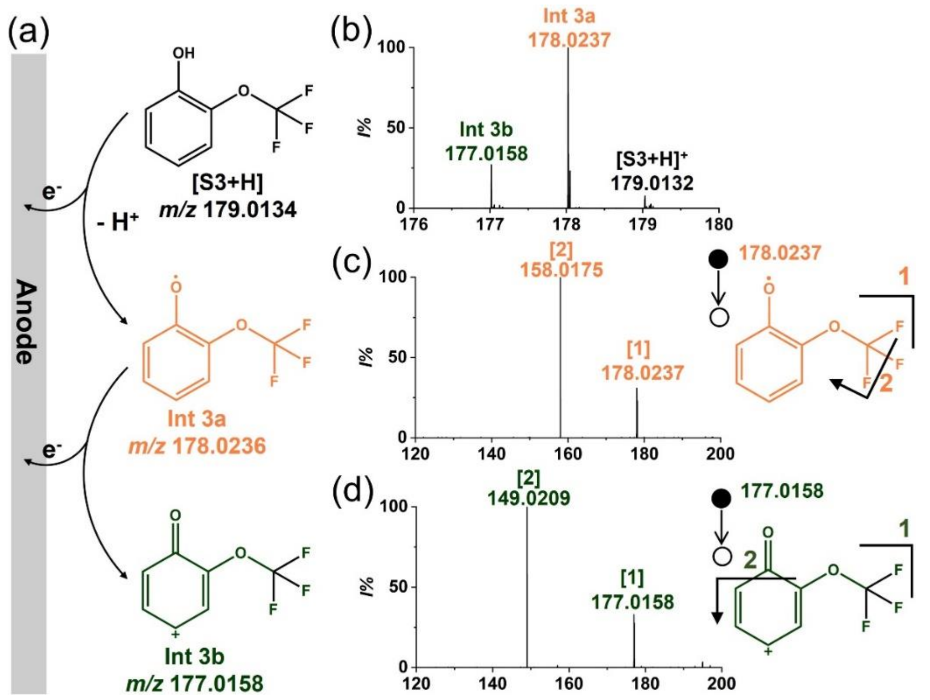

Figure S22. Identification of the reactive intermediates formed upon the electrooxidation of 2-(trifluoromethoxy)phenol (**S3**). (a) Proposed reaction pathway for the electrochemical generation of a carbocation intermediate from **S3**; (b) Mass spectrum of **S3** (1 mM) obtained using a hybrid ultramicroelectrode/ion emitter; (c) Tandem mass spectrum of **Int 3a**, and (d) Tandem mass spectrum of **Int 3b.**

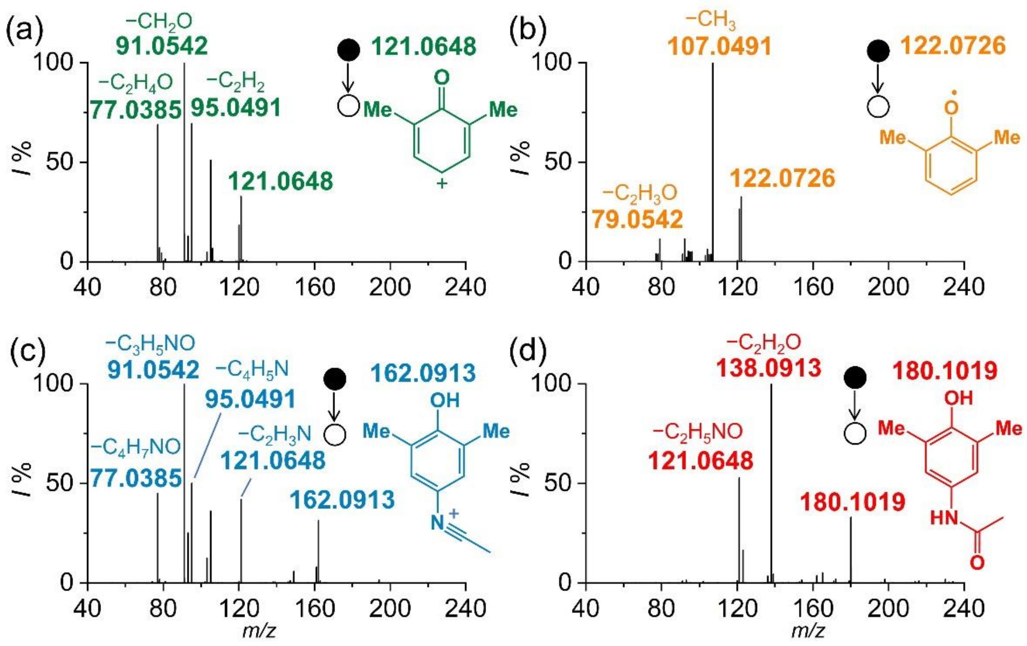

Figure S23. Tandem mass spectrometry of the electrogenerated species upon the anodic oxidation of 2,6-dimethylphenol: (a) the carbocation intermediate, (b) the cationic radical intermediate, (c) the Ritter intermediate and (d) the final acetylation product.

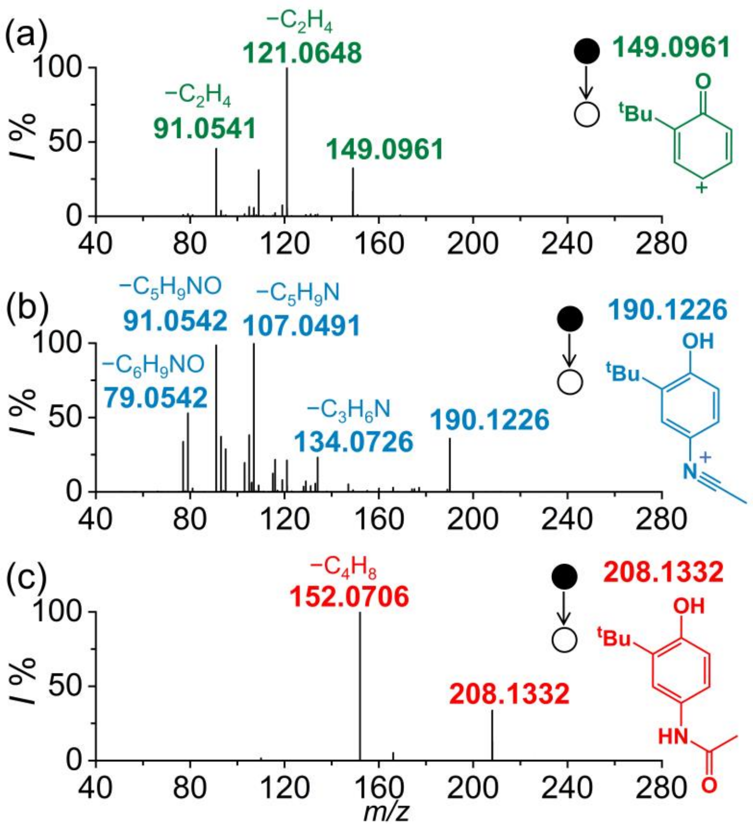

Figure S24. Tandem mass spectrometry of the electrogenerated species upon the anodic oxidation of 2-tert-Butylphenol: (a) the carbocation intermediate, (b) the Ritter intermediate and (c) the final acetylation product.

Table S1. The chemical structures, chemical formulas, theoretical and measured *m/z* values of the 14 benzyl carbocations.

Benzyl carbocations	Chemical formula	Theoretical *m/z*	Measured *m/z*	Error (mDa)
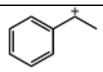	$[C_8H_9]^+$	105.0699	105.0702	0.3
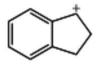	$[C_9H_9]^+$	117.0699	117.0701	0.2
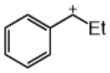	$[C_9H_{11}]^+$	119.0855	119.0857	0.2
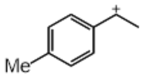	$[C_9H_{11}]^+$	119.0855	119.0856	0.1
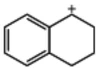	$[C_{10}H_{11}]^+$	131.0855	131.0856	0.1
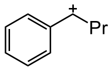	$[C_{10}H_{13}]^+$	133.1012	133.1015	0.3
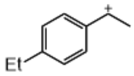	$[C_{10}H_{13}]^+$	133.1012	133.1015	0.3
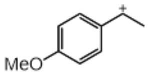	$[C_9H_{11}O]^+$	135.0804	135.0805	0.1
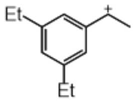	$[C_{12}H_{17}]^+$	161.1325	161.1327	0.2
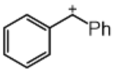	$[C_{13}H_{11}]^+$	167.0855	167.0857	0.2
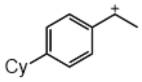	$[C_{14}H_{19}]^+$	187.1481	187.1484	0.3
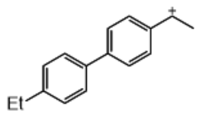	$[C_{16}H_{17}]^+$	209.1325	209.1326	0.1
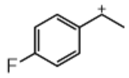	$[C_8H_8F]^+$	123.0605	123.0607	0.2
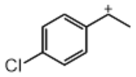	$[C_8H_8Cl]^+$	139.0309	139.0312	0.3

Table S2. The chemical structures, chemical formulas, theoretical and measured *m/z* values of C(sp^2)–H amination products.

Amination products	Chemical formula	Theoretical *m/z*	Measured *m/z*	Error (mDa)
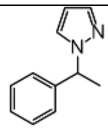	$[C_{11}H_{12}N_2+H]^+$	173.1073	173.1076	0.3
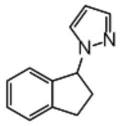	$[C_{12}H_{12}N_2+H]^+$	185.1073	185.1075	0.2
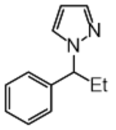	$[C_{12}H_{14}N_2+H]^+$	187.1230	187.1231	0.1
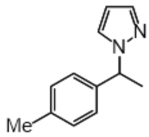	$[C_{12}H_{14}N_2+H]^+$	187.1230	187.1233	0.3
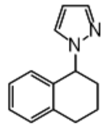	$[C_{13}H_{14}N_2+H]^+$	199.1230	199.1231	0.1
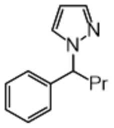	$[C_{13}H_{16}N_2+H]^+$	201.1386	201.1387	0.1
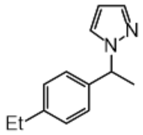	$[C_{13}H_{16}N_2+H]^+$	201.1386	201.1388	0.2
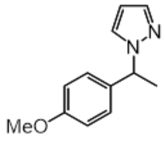	$[C_{12}H_{14}N_2O+H]^+$	203.1179	203.1182	0.3
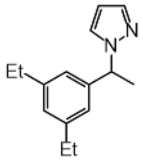	$[C_{15}H_{20}N_2+H]^+$	229.1699	229.1701	0.2
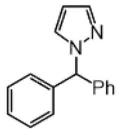	$[C_{16}H_{14}N_2+H]^+$	235.1230	235.1231	0.1
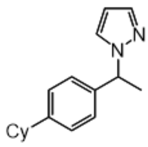	$[C_{17}H_{22}N_2+H]^+$	255.1856	255.1859	0.3

Table S2 (Continued). The chemical structures, chemical formulas, theoretical and measured *m/z* values of C(sp^2)–H amination products.

Amination products	Chemical formula	Theoretical *m/z*	Measured *m/z*	Error (mDa)
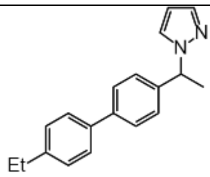	$[C_{19}H_{20}N_2+H]^+$	277.1699	277.1701	0.2
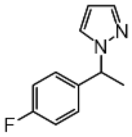	$[C_{11}H_{11}FN_2+H]^+$	191.0979	191.0981	0.2
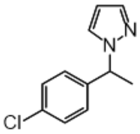	$[C_{11}H_{11}ClN_2+H]^+$	207.0684	207.0687	0.3

Table S3. The chemical structures, chemical formulas, theoretical and measured *m/z* values of product from the electrochemical C–H/N–H cross coupling reactions between 4-ethylbiphenyl and pyrazole derivatives.

Coupling products	Chemical formula	Theoretical *m/z*	Measured *m/z*	Error (mDa)
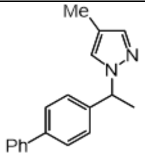	$[C_{18}H_{18}N_2+H]^+$	263.1543	263.1541	-0.2
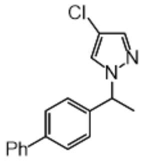	$[C_{17}H_{15}ClN_2+H]^+$	283.0997	283.0995	-0.2
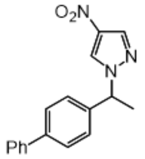	$[C_{17}H_{15}N_3O_2+H]^+$	294.1237	294.1236	-0.1
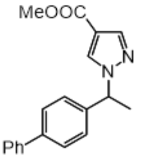	$[C_{19}H_{18}N_2O_2+H]^+$	307.1441	307.1440	-0.1
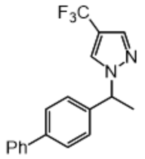	$[C_{18}H_{15}F_3N_2+H]^+$	317.1260	317.1258	-0.2
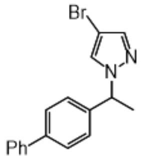	$[C_{17}H_{15}BrN_2+H]^+$	327.0491	327.0490	-0.1

Table S4. The chemical structures, chemical formulas, theoretical and measured *m/z* values of product from the electrochemical C–H/N–H cross coupling reactions between 4-ethylbiphenyl and triazole derivatives.

Coupling products	Chemical formula	Theoretical *m/z*	Measured *m/z*	Error (mDa)
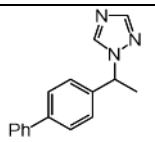	$[C_{16}H_{15}N_3+H]^+$	250.1339	250.1341	0.2
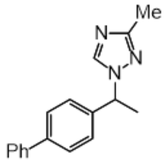	$[C_{17}H_{17}N_3+H]^+$	264.1495	264.1496	0.1
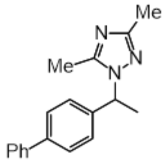	$[C_{18}H_{19}N_3+H]^+$	278.1652	278.1654	0.2
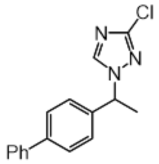	$[C_{16}H_{14}ClN_3+H]^+$	284.0949	284.0952	0.3
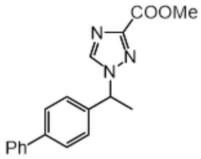	$[C_{18}H_{17}N_3O_2+H]^+$	308.1394	308.1396	0.2
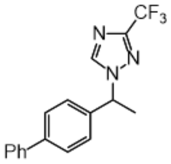	$[C_{17}H_{14}F_3N_3+H]^+$	318.1213	318.1215	0.2

Table S5. The chemical structures, chemical formulas, theoretical and measured *m/z* values of product from the electrochemical C–H/N–H cross coupling reactions between 4-ethylbiphenyl and pyridine derivatives.

Coupling products	Chemical formula	Theoretical *m/z*	Measured *m/z*	Error (mDa)
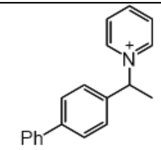	$[C_{19}H_{18}N]^+$	260.1434	260.1435	0.1
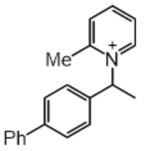	$[C_{20}H_{20}N]^+$	274.1590	274.1592	0.2
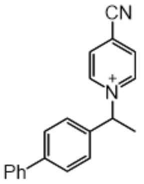	$[C_{20}H_{17}N_2]^+$	285.1386	285.1388	0.2
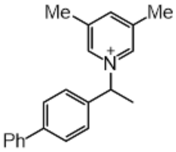	$[C_{21}H_{22}N]^+$	288.1747	288.1748	0.1
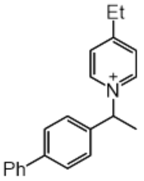	$[C_{21}H_{22}N]+$	288.1747	288.1748	0.1
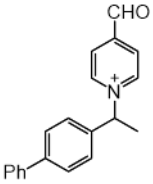	$[C_{20}H_{18}NO]+$	288.1383	288.1386	0.3
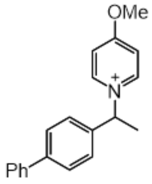	$[C_{20}H_{20}NO]+$	290.1539	290.1542	0.3
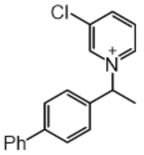	$[C_{19}H_{17}ClN]+$	294.1044	294.1045	0.1
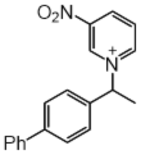	$[C_{19}H_{17}N_2O_2]^+$	305.1285	305.1287	0.2

Table S5 (Continued). The chemical structures, chemical formulas, theoretical and measured *m/z* values of product from the electrochemical C–H/N–H cross coupling reactions between 4-ethylbiphenyl and pyridine derivatives.

Coupling products	Chemical formula	Theoretical *m/z*	Measured *m/z*	Error (mDa)
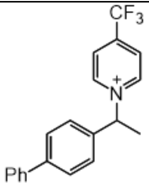	$[C_{20}H_{17}F_3N]^+$	328.1308	328.1311	0.3

Table S6. The chemical structures, chemical formulas, theoretical and measured *m/z* values of product from the electrochemical C–H/N–H cross coupling reactions between 4-ethylbiphenyl and miscellaneous derivatives.

Coupling products	Chemical formula	Theoretical *m/z*	Measured *m/z*	Error (mDa)
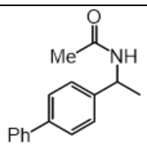	$[C_{16}H_{17}NO+H]^+$	240.1383	240.1385	0.2
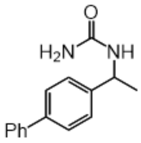	$[C_{15}H_{16}N_2O+H]^+$	241.1335	241.1336	0.1
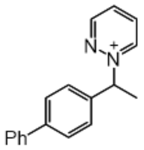	$[C_{18}H_{17}N_2]^+$	261.1386	261.1389	0.3
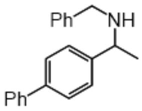	$[C_{21}H_{21}N+H]^+$	288.1747	288.1748	0.1
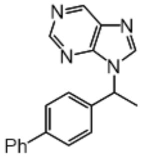	$[C_{19}H_{16}N_4+H]^+$	301.1448	301.1450	0.2
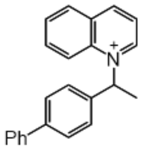	$[C_{23}H_{20}N]^+$	310.1590	310.1591	0.1

Table S7. The chemical structures, chemical formulas, theoretical and measured *m/z* values of the electrogenerated species upon the anodic oxidation of 2,6- dimethylphenol in acetonitrile water.

Electrogenerated species	Chemical formula	Theoretical *m/z*	Measured *m/z*	Error (mDa)
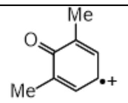	$[C_8H_9O]^{\bullet+}$	122.0726	122.0728	0.2
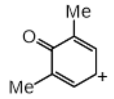	$[C_8H_9O]^{+}$	121.0648	121.0650	0.2
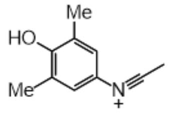	$[C_{10}H_{12}NO]^{+}$	162.0913	162.0915	0.2
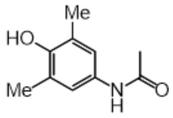	$[C_{10}H_{13}NO_2{+}H]^{+}$	180.1019	180.1021	0.2

Table S8. The chemical structures, chemical formulas, theoretical and measured *m/z* values of the electrogenerated species upon the anodic oxidation of 2-tert-butylphenol in acetonitrile water.

Electrogenerated species	Chemical formula	Theoretical *m/z*	Measured *m/z*	Error (mDa)
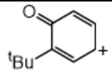	$[C_{10}H_{13}O]^{+}$	149.0961	149.0963	0.2
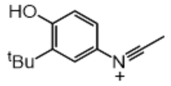	$[C_{12}H_{16}NO]^{+}$	190.1226	190.1228	0.2
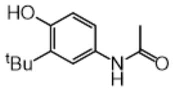	$[C_{12}H_{17}NO_2{+}H]^{+}$	208.1332	208.1334	0.2
